# Rational designing of glyco-nanovehicles to target cellular heterogeneity†

**DOI:** 10.1039/d1sc00140j

**Published:** 2021-01-28

**Authors:** Prashant Jain, Chethan D. Shanthamurthy, Preeti Madhukar Chaudhary, Raghavendra Kikkeri

**Affiliations:** Department of Chemistry, Indian Institute of Science Education and Research Pune-411008 India rkikkeri@iiserpune.ac.in

## Abstract

The aberrant expression of endocytic epidermal growth factor receptors (EGFRs) in cancer cells has emerged as a key target for therapeutic intervention. Here, we describe for the first time a state-of-the-art design for a heparan sulfate (HS) oligosaccharide-based nanovehicle to target EGFR-overexpressed cancer cells in cellular heterogeneity. An ELISA plate IC_50_ inhibition assay and surface plasma resonance (SPR) binding assay of structurally well-defined HS oligosaccharides showed that 6-*O*-sulfation (6-*O*-S) and 6-*O*-phosphorylation (6-*O*-P) of HS tetrasaccharides significantly enhanced EGFR cognate growth factor binding. The conjugation of these HS ligands to multivalent fluorescent gold nanoparticles (AuNPs) enabled the specific and efficient targeting of EGFR-overexpressed cancer cells. In addition, this heparinoid-nanovehicle exhibited selective homing to NPs in cancer cells in three-dimensional (3D) coculture spheroids, thus providing a novel target for cancer therapy and diagnostics in the tumor microenvironment (TME).

## Abstract 2

Heparan sulfate oligosaccharide based nanovehicle greatly enhance the selective targeting of cancer cells in tumor microenvironment.

## Graphical abstract

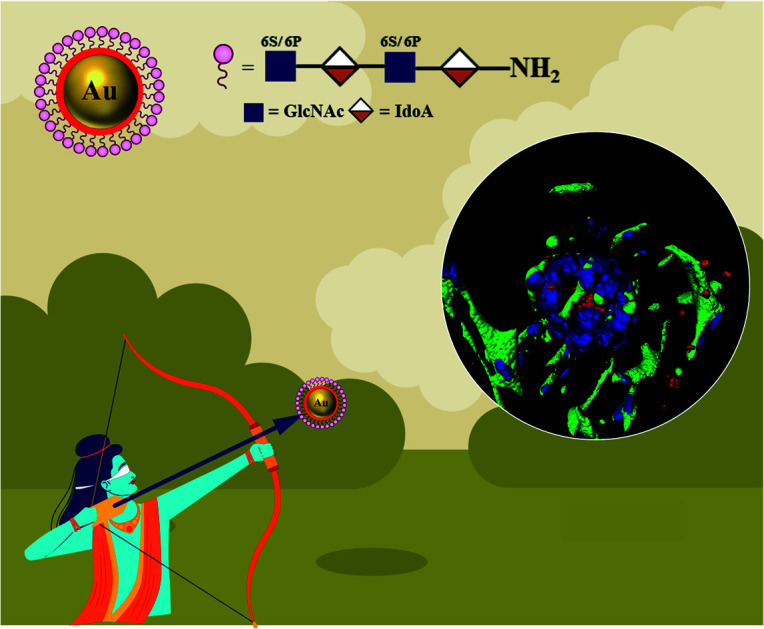

## Introduction

Cancer is a devastating and multifactorial disease. Recent studies have confirmed that over-expression of certain cell-surface receptors, and growth factors, and suppression of tumor-specific genes are primary causes of cancer phenotype development.^1^ Therefore, developing suitable markers for these functional changes may provide new diagnostic tools and lead to new delivery systems. Over the past two decades, epidermal growth factor receptors (EGFRs) have emerged as potential oncogenes that are commonly found in various cancer types.^2^ Thus, EGFR targeted small molecules and EGFR-neutralizing monoclonal antibodies have impressive clinical significance.^3^ EGFRs bind to and are activated by their autocrine growth factors, such as EGF and heparin-binding EGF-like growth factors (HB-EGFs) that are often governed by heparan sulfate (HS), which is ubiquitous on both cell surfaces and the extracellular matrix.^4^ Hence, deciphering the structure–function relationship between HB-EGF or EGF and HS could be a new tool for selectively targeting cancer cells in tumor microenvironments.

Structurally, HS is composed of α(1–4)-linked disaccharide repeating units of d-glucosamine and hexuronic acid, which could be either d-glucuronic acid (GlcA) or l-iduronic acid (IdoA).

The structural diversity of HS comes from the degree of *O*-sulfation and *N*-sulfation/acetylation on the glucosamine and hexuronic acid ligands.^5^ Several research groups have synthesized broad well-defined HS oligosaccharide libraries to elucidate the active ligand for growth factors, chemokines and biologically active molecules.^6^ The majority of these HS libraries are able to determine the sulfation code, uronic acid composition and oligosaccharide lengths of the HS during protein recognition. However, to the best of our knowledge, it is still unclear as to what is the HS chemically defined epitope(s) of EGFR specific growth factors.

**Fig. 1:**
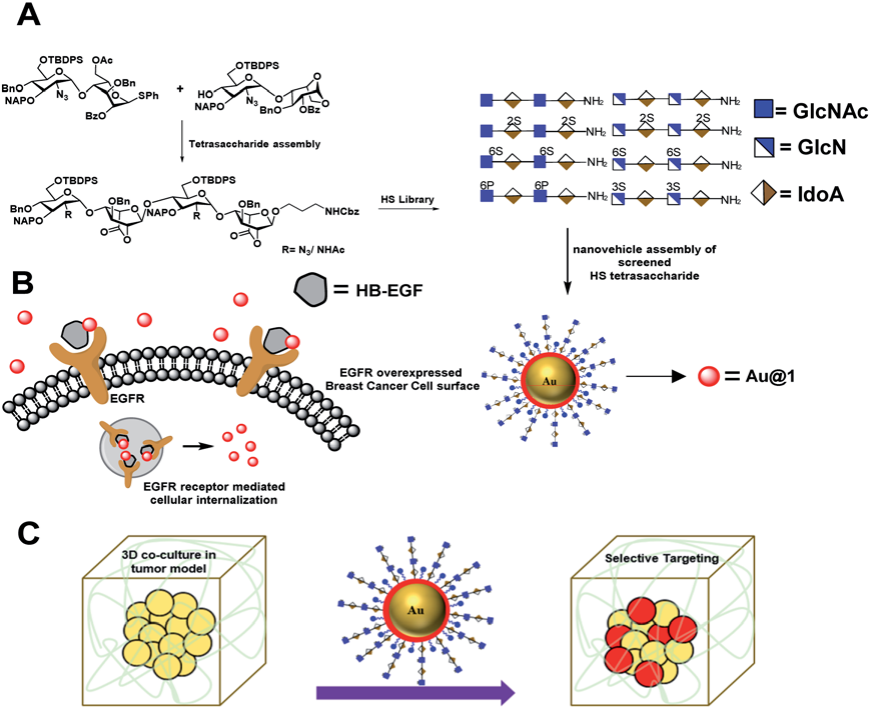
(A) Synthetic heparan sulfate based nanovehicle assembly and cancer cell targeting *via* EGFR in 2D and 3D-coculture models. (B) EGFR mediated uptake of the nanovehicle by breast cancer cells. (C) Schematic representation of the tumor model and selective targeting of cancer cells in the presence of stromal cells and an extracellular matrix.

Herein, we report a new set of HS-tetrasaccharides with well-defined sulfation codes to determine the structure-functional relationship between EGFR binding growth factors. An ELISA assay with HB-EGFs and EGFs rationalized the molecular code of HS for EGFR activation. SPR binding confirms the strength of HS binding affinity. We have also synthesized the phosphate analog of super-active HS-tetrasaccharides in order to determine why nature prefers the sulfate pattern of HS during molecular recognition. Active ligands were functionalized on fluorescent gold nanoparticles to highlight the endocytosis process in different cancer cell lines with variable EGFR expressions. Finally, we developed a three-dimensional coculture spheroid model to demonstrate selective targeting of cancer cells in the presence of stromal cells and the extracellular matrix (Fig. 1).

Although HS/heparin polymer-based nanoparticles targeting cancer cells have been reported in the literature,^7^ however, structure heterogeneity of the HS polymer rendered native ligands less specific to cancer cells. Here, we report the first example of well-defined HS oligosaccharide-based nanovehicle construction to target specific cancer cells, followed by a 3D-coculture assay to establish selective targeting of cancer cells in the tumor microenvironment.

## Results and discussions

**Scheme 1:**
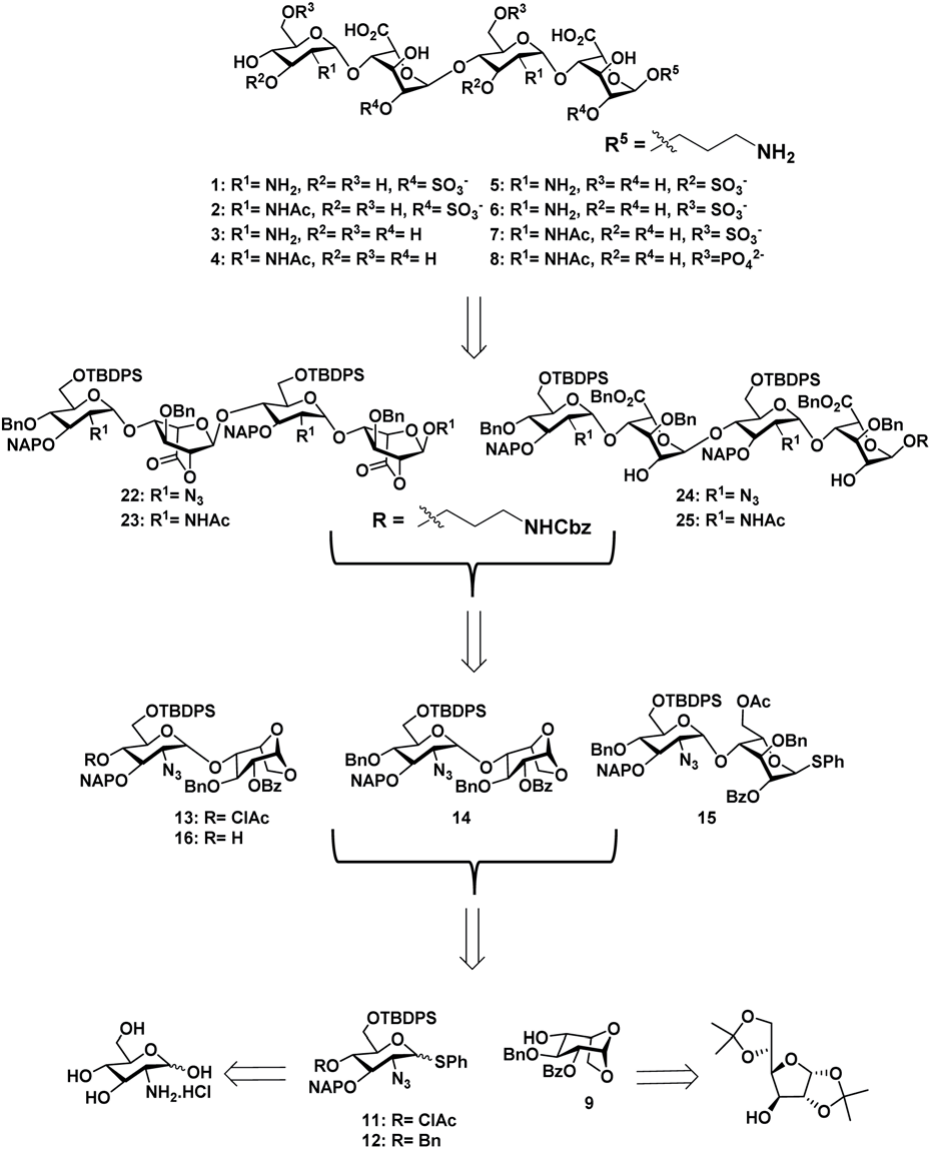
Retrosynthesis of HS tetrasaccharide.

With the aim of identifying HS oligosaccharide ligands for EGFR specific growth factors, we synthesized *N*-unsubstituted and *N*-acetate derivatives of glucosamine with sulfation substitution at 6-OH, 3-OH and 2-OH of iduronic acid residue respectively. The rationale behind this sulfation patterns is that they exhibit a common binding pocket for various growth factors and chemokines. We adopted the [2 + 2] glycosylation strategy comprising glycosyl donor **15** and acceptor **16** to synthesize tetrasaccharide precursors **22** and **23** (Scheme 1). The disaccharide assemblies **13** and **14** (ref. 8) were obtained from orthogonally protected d-glucosamine donors **11** and **12** and acceptor l-idopyranosyl **9** (ref. 9) under standard glycosylation conditions. The thiodonor **11** carries C-4 chloroacetyl and C-6 silyl as a facile protecting group which can be removed easily at the later stage for further modification and chain elongation. The 4-*O*-chloroacetate of **13** was selectively deprotected in the presence of thiourea to afford acceptor **16** in quantitative yield. The disaccharide donor **15** was obtained by acetolysis of **14** using acetic anhydride and copper(ii) trifluoromethanesulfonate as a catalyst followed by phenyl trimethylsilyl sulphide and ZnI_2_ treatment to generate corresponding thioglycoside in excellent yield.^10^ Finally, glycosylation of donor **15** and acceptor **16** by utilizing *N*-iodosuccinimide (NIS) and the trimethylsilyl trifluoromethanesulfonate (TMSOTf) promoter resulted in 90% of tetrasaccharide **17** (Scheme 1).

**Scheme 2:**
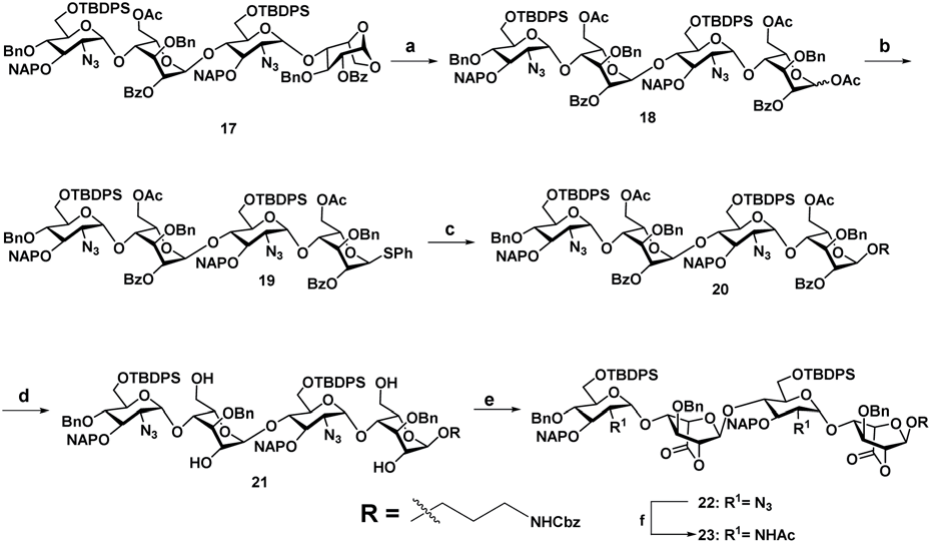
(a) Ac_2_O, Cu(OTf)_2_, rt, 12 h, 88%. (b) TMSSPh, ZnI_2_, CH_2_Cl_2_, rt, 2 h, 86%. (c) Benzyl(3-hydroxypropyl)carbamate, NIS, TfOH, 4 Å MS, rt, CH_2_Cl_2_, 30 min, 84%. (d) NaOMe, CH_2_Cl_2_/MeOH (1 : 1), rt, 12 h, 86%. (e) TEMPO, CH_2_Cl_2_/MeOH (1 : 1), rt, 12 h, 95%. (i) Zn, THF/AcOH/Ac_2_O (3 : 2 : 2), rt, 12 h, 72%.

**Scheme 3:**
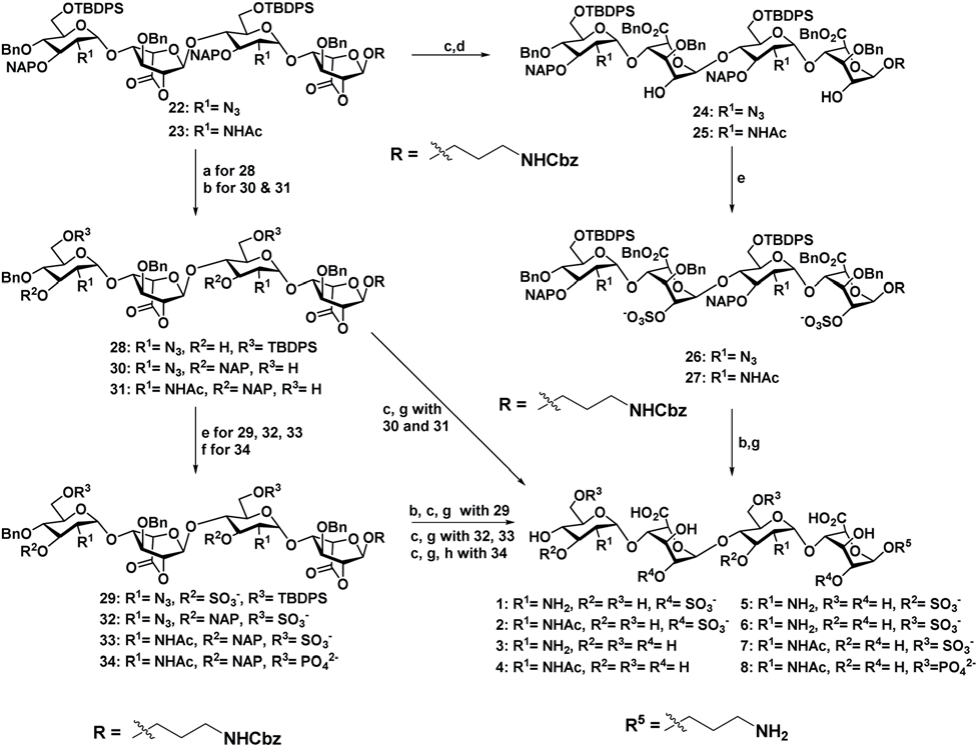
(a) DDQ, CH_2_Cl_2_/H_2_O (18 : 1), rt, 1 h, 53%. (b) 70% HF·py, py, 0 °C, 12 h, (**30**, 83%, and **31**, 87%). (c) LiOH·H_2_O, THF/H_2_O (1 : 1), rt, 2 h. (d) BnBr, TBAI, NaHCO_3_, DMF, 60 °C, 2 h, (**24**, 75%, and **25**, 73% for two steps). (e) SO_3_·NEt_3_, DMF, 60 °C, 72 h, (**26**, 63%; **27**, 51%; **29**, 52%; **32**, 70%; **33**, 65%). (f) DPPC, DMAP, NEt_3_, CH_2_Cl_2_/py (1 : 1), 0 °C, 12 h, 60%. (g) H_2_, Pd(OH)_2_, MeOH, rt, 36 h, [**1**, 75%; **2**, 81%, **3**, 72% (two steps); **4**, 68% (two steps); **5**, 50% (three steps) **6**, 72% (two steps); **7**, 70% (two steps)]. (h) H_2_, PtO_2_, MeOH, rt, 24 h, 45% (three steps).

Sequential acetolysis, thiophenol glycosylation and linker glycosylation of **17** at the reducing end of IdoA resulted in **20** in a three step yield of 63% (Scheme 2). Successive deacetylation of **20** and oxidation of primary alcohol with a catalytic 2,2,6,6-tetramethyl-1-piperidinyloxyl free radical (TEMPO) and [bis(acetoxy)iodo]benzene (BAIB) delivered the lactonized HS-tetrasaccharide precursor **22**. The C-2 azide of **22** was converted to acetamide in the presence of Zn/AcOH/Ac_2_O yielding 72% of **23**. Using the divergent synthetic strategy, **22** and **23** were converted into desired HS tetrasaccharide using selective or global deprotection and the sulfonation strategy (Scheme 3). Briefly, one-pot lactam ring opening by using LiOH and, benzyl esterification of **22** and **23** yielded **24** and **25** respectively, which were sulfated in the presence of SO_3_·Et_3_N in DMF to yield 2-*O*-sulfated HS tetrasaccharide precursors **26** and **27**. Similarly, selective deprotection of NAP and TBDPS by using DDQ and 70% HF·py respectively afforded 3-*O* and 6-*O*-hydroxyl derivatives. 3-*O*-sulfation of **28** and 6-*O*-sulfation of **30** and **31** yielded 50–75% of **29**, **32** and **33**. Finally, global deprotection by lactam-ring opening and hydrogenolysis yielded desired **1–7** HS-tetrasaccharides (Scheme 3).

**Fig. 2:**
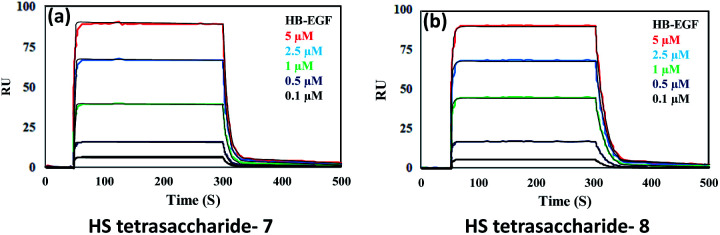
SPR binding analysis for the interaction between the HB-EGF and (a) HS tetrasaccharide **7**; (b) HS tetrasaccharide **8**.

Next, we confirmed the HB-EGF binding affinity of **1–7** HS-tetrasaccharides. To this end, native heparin was immobilized on an ELISA plate and standard competition assay with heparinoids (**1–7**) was performed using HB-EGF, and EGF proteins. ELISA analysis revealed that the only 6-*O*-S HS tetrasaccharide (**7**) showed strong inhibition with HB-EGF binding to native HS (IC_50_ = 126.6 μM) (Fig. S2†) compared to 2-*O*-S, 3-*O*-S and non-sulfated HS-tetrasaccharide analogs (**1–6**) at a concentration between 0 and 1 mg l^−1^. In contrast, EGF protein showed no binding with synthetic HS-tetrasaccharides. An additional surface plasmon resonance (SPR) binding experiment of **7** with HB-EGF revealed a *K*_D_ of 11.12 μM (Fig. 2a and Table S1†), clearly illustrating that the 6-*O*-S HS tetrasaccharide functioned as an active ligand for the HB-EGF. To determine whether comp. **7** and HB-EGF interactions differentiate between charge species geometrically close to the sulfate group,^11^ we synthesized 6-*O*-phosphated HS tetrasaccharide by phosphorylating **31** using diphenyl phosphoryl chloride to yield 60% of **34** (Scheme 3). Finally, LiOH mediated lactone ring opening and hydrogenolysis yielded 45% of **8** (Scheme 3). The ELISA assay and SPR binding showed strong binding with the HB-EGF (IC_50_ = 97.13 μM) (Fig. S2†) and a *K*_D_ of 11.06 μM (Fig. 2b and Table S1†). These results demonstrated that the sulfated and phosphated HS tetrasaccharides functioned as active ligands of the HB-EGF. It would be interesting to determine whether both ligands target cancer cells by activating the EGFR through autocrine HB-EGF signaling.

**Scheme 4:**
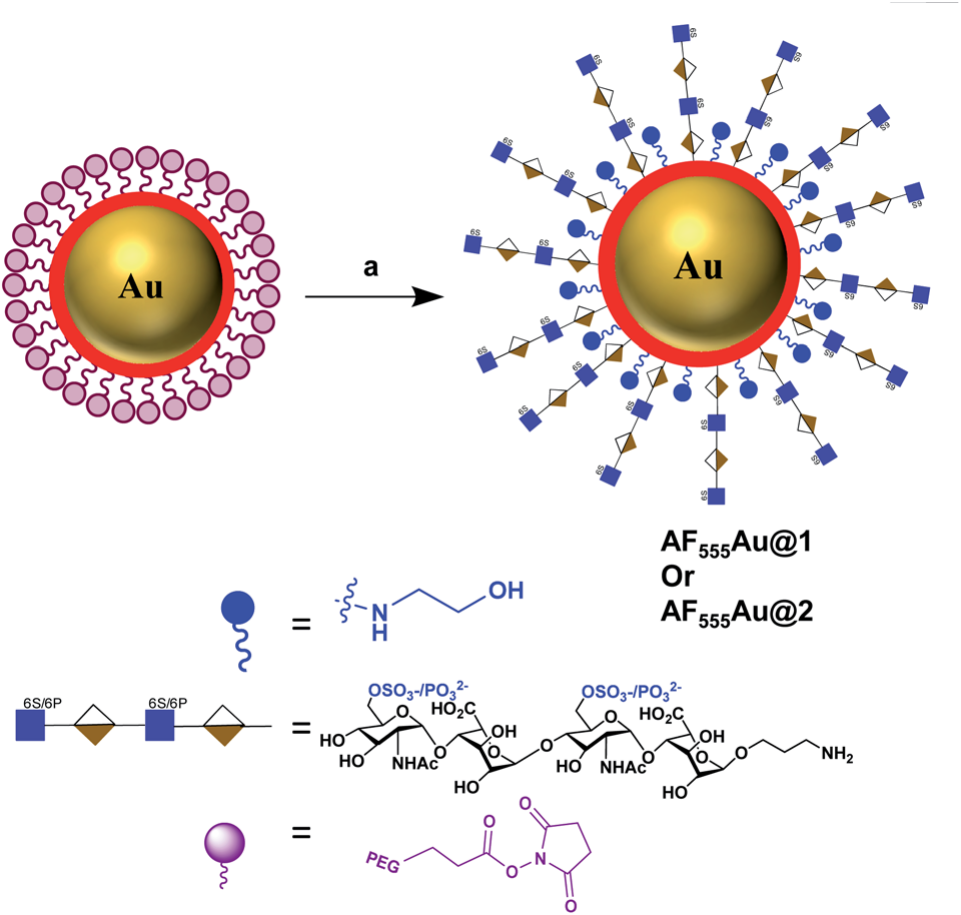
Synthesis of heparinoid AuNPs: reagents and conditions: (a) **7** or **8**, PBS, rt, 12 h.

To assess the efficacy of the HS-tetrasaccharide ligands in activating EGFRs, we functionalized them using commercial *N*-hydroxysuccinimide-active fluorescent AuNPs (AF_555_Au), which served as optical and non-toxic probes.^12^ The nanoparticle functionalization was performed by mixing ligands **7** and **8** at RT in 0.01 M PBS buffer with a pH of 7.5 for 12 h (Scheme 4). The remaining NHS groups were then neutralized with ethanolamine to afford heparinoid-capped fluorescent AuNPs (**AF555Au@1** and **AF555Au@2**), collectively represented as heparinoid-AuNPs. The physical properties of **AF555Au@1** and **AF555Au@2** were confirmed by transmission electron microscopy (TEM), UV-visible and fluorescence spectroscopy and zeta potential measurements (Table S2, Fig. S3 and S4†). As a control, native heparan sulfate was conjugated with Texas Red (T-HP) and characterized (Fig. S1†).

**Fig. 3:**
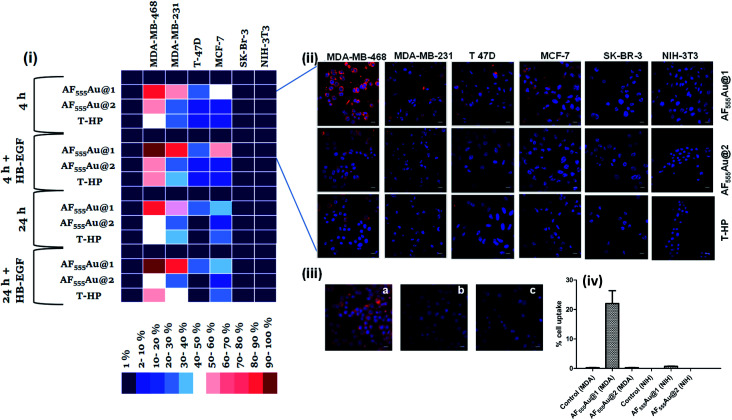
(i) Hierarchical clustering analysis (HCA) of cellular internalization of heparinoid-AuNPs with different breast cancer cells and normal cells after 4 and 24 h in the presence and absence of EGF proteins (HB-EGF mediated uptake of **AF555Au@1** after 24 h was considered as 100%); (ii) confocal images of nanoparticle internalization by different cell lines after 4 h (scale bar: 20 μm); (iii) confocal images of **AF555Au@1** in the presence of different endocytotic pathway inhibitors (scale bar: 20 μm): (a) **AF555Au@1**; (b) NaN_3_ + **AF555Au@1**; (c) EGF inhibitor + **AF555Au@1**. (iv) (c) Quantification of the cellular uptake in MDA-MB-468 and NIH-3T3 for **AF555Au@1** and **AF555Au@2** using flow cytometry.

Next, a cellular uptake assay was performed using the standard protocol described in the ESI.† Breast cancer cell lines were selected based on the EGFR expression level (MDA-MB-468 high degree; MDA-MB-231, T-47D and MCF-7: moderate to low degree; and SK-BR-3 least EGFR expression^13^). The cancer cells and NIH-3T3 (as normal cells) were seeded on eight-well glass chamber slides and allowed to grow until they reached 70–80% confluency at 37 °C in a 5% CO_2_ incubator. Heparinoid-AuNPs (15 μg ml^−1^) and T-HP (10 μg ml^−1^) were added to the wells, and live images were recorded at two different time intervals (4 h and 24 h) (Fig. 3(ii) and S5†). To demonstrate the HB-EGF-mediated uptake, the uptake mechanism was tested in the presence of 0.1 ng ml^−1^ of the HB-EGF proteins (Fig. S6†). Hierarchical clustering (HCA) was developed based on the fluorescence intensity of the heparinoid-AuNPs inside the cells (Fig. 3(i)). To ensure the consistency of the findings, all tests were performed in triplicate. The HCA of the heparinoid-AuNP cellular internalization assay indicated the presence of a distinct disparity in uptake rates. As expected, the MDA-MB-468 cell line showed stronger cellular internalization responses than the other breast cancer cell lines and normal cells. Among the heparinoids, the uptake rate of **AF555Au@1** was approximately 70–80% stronger after 4 h (Fig. 3(ii)), and the uptake rate significantly increased in the presence of the HB-EGF protein (Fig. S6†). This trend continued after 24 h. MDA-MB-468 showed a preferential uptake of the sulfated-heparinoid over the phosphate analog, confirming the critical role of 6-*O*-sulfation of glucosamine in HB-EGF/EGFR signaling.

The uptake rate in the other breast cancer cell lines was weak as compared to that of MDA-MB-468. Moreover, T-HP exhibited a weak cellular uptake rate when compared to the heparinoid-AuNPs, indicating that synthetic ligand **7** might be a better functional ligand in terms of targeting the EGFR than the native HS sequence. We also performed FACS assays with MDA-MB-468 and NIH-3T3 to quantify the percentage cell uptake of the heparinoid-AuNPs. FACS analysis clearly revealed the potential uptake of **AF555Au@1** in MDA-MB-468 and no uptake in the normal NIH-3T3 cells (Fig. 3(iv)). On the basis of these results, we hypothesized that **AF555Au@1** could serve as a potential nanovehicle for targeting breast cancer cells.

To elucidate the mechanism underlying endocytosis, we performed live confocal imaging studies in the presence of endocytic pathway inhibitors. First, we evaluated the energy-dependent pathway. To this end, we incubated MDA-MB-468 with sodium azide so as to deplete the ATP and then administered the **AF555Au@1** treatment for 4 h. We observed a substantial decrease in cellular internalization of the nanoparticles, indicating the presence of receptor-mediated endocytic pathways (Fig. 3(iii-b)). To analyze EGFR-mediated endocytosis, gefitinib (an EGFR inhibitor) (30 μM) was added. The blockage of the receptor resulted in a strong decrease in the cellular uptake of **AF555Au@1** (Fig. 3(iii-c)). These findings suggested that **AF555Au@1** undergoes receptor-mediated endocytosis.

**Fig. 4:**
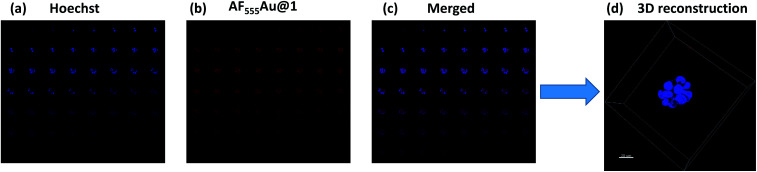
Z-stack montage for MDA-MB-468 spheroids (total slices: 52); (a) nuclei staining (blue channel); (b) **AF555Au@1** (red channel); (c) merged (blue and red); (d) 3D reconstruction of the z-stacked images (scale bar: 20 μm).

Recent research established that a two-dimensional (2D) monolayer cell assay did not replicate the *in vivo* tumor model to evaluate the efficacy of nanovehicles in cancer therapy.^14^ Alternatively, three-dimensional (3D) spheroids can provide an attractive *in vitro* model that accurately mimics the tumor microenvironment (TME) for the purposes of drug discovery and tumor targeting.^15^ The TME comprises tumor cells encapsulated by a dense extracellular matrix (ECM) as well as heterogeneous cell types such as stromal cells, immune cells, and endothelial cells. These heterogeneous cellular environments, together with the composition of the ECM, support the enhancement of cancer cell motility, activate different signaling pathways, and reduce the targeting efficacy of nanovehicle delivery to the cancer cells.^16^ Thus, it is important to examine the activity of heparinoid-AuNPs in a 3D-spheroid model to demonstrate the efficacy. We first constructed MDA-MB-468 and SK-BR-3 cell microspheroids using ECM Matrigel. After eight days of culture, we observed the formation of spheroids that were uniform in size (50–52 μm) and contained approximately 10–15 cells per spheroid. We added **AF555Au@1** at an optimum concentration of 50 μg ml^−1^. Confocal imaging revealed a strong uptake of **AF555Au@1** by the MDA-MB-468 cells (Fig. 4). However, no uptake of **AF555Au@1** was observed in the SK-BR-3 cells. Well-organized spheroids composed of hoechst stained nuclei (Fig. 4a) and the red fluorescent **AF555Au@1** (Fig. 4b) were co-localized as shown by a 3D reconstruction of the confocal Z-stack images (Fig. 4c and d). These results confirmed that **AF555Au@1** diffused into the ECM and targeted the MDA-MB-468 cells.

**Fig. 5:**
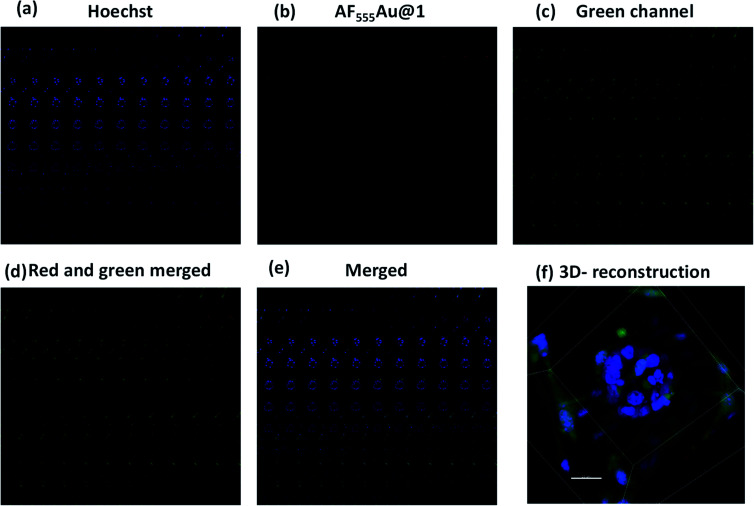
Z-stack montage for MDA-MB-468 and GFP stable NIH-3T3 cell spheroids (total slices: 106); (a) nuclei stain (blue channel); (b) **AF555Au@1** (red channel); (c) GFP-NIH-3T3 (green channel); (d) merged (red and green); (e) merged (blue, red and green); (f) 3D reconstruction of Z-stacked images (scale bar: 25 μm).

Next, we investigated whether the cancer cells were selectively targeted in the presence of stromal cells such as fibroblast cells. Fibroblast cells are one of the prominent cell types in the TME, and they play a pivotal role in ECM remodeling and inducing resistance to the uptake mechanism of nanovehicles by cancer cells.^17^ To shed light on selective targeting of cancer cells in the presence of stromal cells, we constructed 3D co-culture models by mixing an optimum 2 : 1 concentration of MDA-MB-468 and GFP stable NIH-3T3, which also replicate the TME.^18^ After five days, we observed the presence of multicellular spheroids. These spheroids were much larger in size then the MDA-MB-468 alone, and they were composed of 15–20 cells per spheroid with a maximum size of 100–110 μm. In addition, as shown in the Z-stack images (Fig. 5b–d) NIH-3T3 encapsulated the cancer cell spheroids thereby, creating a fibroblast layer outside the tumor cells. To these co-culture models, we added **AF555Au@1** (50 μg ml^−1^). After 4 h, as shown in merged Z-stack images and 3D reconstruction (Fig. 5d and f, respectively), **AF555Au@1** successfully crossed the fibroblast layer and targeted only the cancer cells. These results suggest that **AF555Au@1** targeted the cancer cells in the presence of fibroblast cells, thus demonstrating the efficacy of the nanovehicle in targeting cancer cells in the TME.

## Conclusions

In conclusion, we synthesized a structurally well-defined HS library using a divergent strategy. The 6-*O*-S HS tetrasaccharide was the most active ligand among the series when the binding affinity with EGFR cognate growth factors was tested. This study also confirmed that 6-*O*-phosphate is a potential ligand of HB-EGFs. Using these two ligands, we have constructed HS-based fluorescent-nanovehicles that are intended to target breast cancer cells in tumor microenvironments. Confocal imaging studies confirmed the enhanced uptake of 6-*O*-sulfated HS-tetrasaccharide by EGFR-overexpressed cancer cells, whereas their phosphate derivatives showed weak EGFR-mediated uptake rates. These results confirmed the critical role of HS sulfation groups in EGFR activation. The HS-nanoparticles targeted the breast cancer cells *via* HB-EGF/EGFR-mediated interactions in both the 2D-monolayer and in the 3D-complex coculture tumor model. Overall, these results represent a major step forward toward designing a HS-based nanovehicle for targeted cancer therapies.

## Conflicts of interest

There are no conflicts of interest to declare.

## Supplementary Material

SC-012-D1SC00140J-s001
