# Supplementary material for: Rational designing of glyco-nanovehicles to target cellular heterogeneity
Source: Chem Sci. 2021 Jan 28;12(11):4021–7. doi: 10.1039/d1sc00140j (PMC8179433; doi:10.1039/d1sc00140j)
Supplement: SC-012-D1SC00140J-s001 [file SC-012-D1SC00140J-s001.pdf]

Electronic Supplementary Material (ESI) for Chemical Science.
This journal is © The Royal Society of Chemistry 2021

Supporting Information

Rational Designing of Glyco-nanovehicles to Target Cellular Heterogeneity

Prashant Jain,[a,b] Chethan D. Shanthamurthy,[a,b] Preeti Madhukar Chaudhary[a] and Raghavendra Kikkeri*[a]

[a] Mr. P.Jain, Mr. C. D. Shanthamurthy, Dr. P. M. Chaudhary, Dr. R. Kikkeri

Indian Institute of Science Education and Research

Dr. Homi bhabha road,

Pune-411008, India

E-mail: rkikkeri@iiserpune.ac.in

[b] Equal Contribution

Table of contents

1. General information
2. General Procedures
3. Synthesis of heparinoid oligosaccharides
4. Synthesis and characterization of Texas red-heparin (T-HP)
5. ELISA protocol
6. SPR Analysis
7. Synthesis of heparinoid-gold nanoparticles
8. TEM, UV-visible and Fluorescence measurement of heparinoid-AuNPs
9. Zeta potential measurements
10. Cell viability assay
11. Cellular internalization of heparinoid-AuNPs
12. FACS analysis

13. Co-localization

14. Cellular internalization mechanism studies

15. 3D spheroid formation.

16. Abbreviations

17. References

18. ^{1}H-NMR and ^{13}C-NMR

1. General information

All chemicals were reagent grade and used as supplied except where noted. Analytical thin layer chromatography (TLC) was performed on Merck silica gel 60 F254 plates. Compounds were visualized by UV irradiation or dipping the plate in CAM/ninhydrin solution followed by heating. Column chromatography was carried out using force flow of the indicated solvent on Fluka Kieselgel 60 (230–400 mesh). ^{1}H and ^{13}C NMR spectra of all compounds were recorded on Jeol 400 MHz, and Bruker 600 MHz with cryo probe using residual solvents signals as an internal reference ($CDCl_3$ δH, 7.26 ppm, δC 77.3 ppm, CD_3OD δH 3.31 ppm, δC 49.0 ppm and D_2O δH 4.79 ppm). The chemical shifts (δ) are reported in ppm and coupling constants (J) in Hz. UV-visible measurements were performed with Evolution 300 UV-visible spectrophotometer (Thermo Fisher Scientific, USA). Fluorescence spectra was recorded in FluoroMax-4 spectrofluorometer (Horiba Scientific, U.S.A.). All the confocal microscopy images were captured on a Zeiss LSM710 confocal microscope and processed using ImageJ software. Human HB-EGF, amphiepiregulin, EGF and its respective biotinylated antibody was purchased from Peprotech. Gefitinib (EGFR inhibitor) was purchased from Sigma-aldrich. GFP stable NIH-3T3 cells were purchased from Biogenuix.

2. General Procedures.

Heparin tetrasaccharide **1-8** were synthesized as described previously[1].

General procedure for glycosylation: Solution of donor (1 mmol) and acceptors (0.9 mmol) were dissolved in CH_2Cl_2 and kept for stirring under N_2 atmosphere before the addition of NIS (1.6 mmol) and TMSOTf/ TfOH (0.3 mmol) at desired temperature. Upon completion the reaction was quenched with few drops of Et_3N and filtered through celite pad. The filtrate was washed washed with aqueous $Na_2S_2O_3$ and brine. Finally, the organic layer was concentrated and purified with silica gel column chromatography.

General procedure for Benzyl Ester formation: Solution of starting material (1 mmol) in THF/ H_2O (1/ 1) was stirred with LiOH• H_2O (3 mmol) at room temperature for 2 h. After completion of the reaction it was quenched using Amberlite IR 120H^+ resin then filtered, evaporated and dried. The as obtained residue was dissolved in DMF and stirred along with BnBr (4 mmol), TBAI (1.4 mmol), $NaHCO_3$ (5 mmol) at 60 ^{o} C for another 2 h under N_2 atmosphere. After 2 h, the reaction mixture was extracted with ethyl acetate, washed with brine and, purified through silica gel column chromatography.

General procedure for acetolysis: The solution of starting material (1 mmol) in acetic anhydride (10 ml for 1g) was stirred at 0 ^oC- rt in the presence of $Cu(OTf)_2$ (0.1 mmol) and left for stirring at room temperature for 12 h. After completion of reaction, acetic anhydride was evaporated and washed with saturated $NaHCO_3$ solution before purifying through silica gel chromatography.

General procedure for anomeric thiophenol donor preparation: The solution of starting material (1 mmol) in CH_2Cl_2 was stirred along with ZnI_2 (2 mmol) and phenyl trimethylsilyl sulphide (3 mmol) for 2 h. After that, the reaction mixture was filtered through celite pad and concentrated under reduced pressure before purifying through silica gel column chromatography.

General procedure for desilylation pre *O*- sulfation: Solution of starting material (1 mmol) in pyridine (2 mL) was stirred along with 70 % HF.py complex (5 mmol) at ice cold temperature under N_2 atmosphere for 12 h. Addition of HF.py complex was carried out in drop wise manner. Upon formation of the product, the mixture was extracted with ethyl acetate and washed with 1 N HCl and brine. The combined organic layer was dried over Na_2SO_4, filtered, concentrated and purified through silica gel column chromatography.

General procedure for *O*-Sulfation: Solution of starting material (1 mmol) in DMF (2 mL) was stirred along with $SO_3.NEt_3$ complex (10 mmol) under N_2 atmosphere at 60 ^{o} C for 3 days. After completion of the reaction, it was quenched with aqueous $NaHCO_3$ (15-20 mmol) while on stirring for another 16 h. Finally, the reaction mixture was concentrated under reduced pressure and the resulting residue was filtered with MeOH through wattman filter paper. The filtrate was concentrated and purified using silica column chromatography using CH_2Cl_2/ MeOH as solvents.

General procedure for desilylation post *O*- sulfation: The solution of starting material (1 mmol) in pyridine (2 mL) and HF.py (5 mmol) was stirred at 0 ^{o}C- rt for 12 h. After that the volatiles were evaporated and the residue was purified through Sephadex LH 20 column using MeOH as a eluent.

General procedure for global deprotection:

Lactone ring opening: The solution of starting material (1 mmol) in THF (1 mL) and H_2O (1 mL) along with $LiOH.H_2O$ (10 mmol) was stirred at room temperature for 2 h. Upon completion, the reaction mixture was diluted with MeOH and quenched using Dowex 50WX8 H^+ resin. The mixture was filtered, concentrated and passed through Bond Elute C-18 column. The combined fraction was concentrated under reduced pressure and dissolved in MeOH for hydrogenolysis.

Hydrogenolysis: The reaction mixture along with $Pd(OH)_2$ was stirred under H_2 atm for 36 h. After that, the mixture was filtered, concentrated and eluted through Bond Elute C-18 column using H_2O as eluent. The combined H_2O fraction were pooled and lyophilized to yield fully deprotected *O*- sulfated tetrasaccharides. For **8** after $Pd(OH)_2$ hydrogenolysis reaction mixture was again hydrogenolysis using PtO_2 as a catalyst under H_2 atm for 24 h for the removal of phenyl group. After 24 h the reaction mixture was filtered and eluted through Bond Elute C-18 column using H_2O as eluent. The combined H_2O fraction were pooled and lyophilized to yield fully deprotected 6-*O*- phosphorylated tetrasaccharide **8.**

3. Synthesis of heparinoid oligosaccharides (1-8).

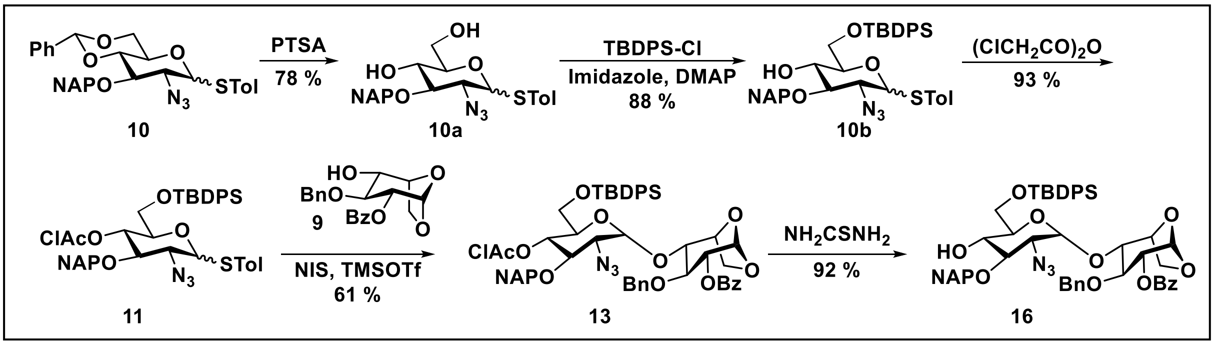

Compound 11

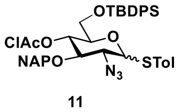

Synthesis of **11** was carried out from previously reported molecule **10**. First, benzylidene ring from **10**[1] (1 mmol) was cleaved using PTSA (1.5 mmol) in MeOH. Upon completion of the reaction, the mixture was quenched using Amberlite IR $120H^+$, filtered, concentrated and purified through flash column chromatography. Next, 6-*O* hydroxy of **10a** (1 mmol) was protected using TBDPS chloride (1.2 mmol) in the presence of DMAP (0.2 mmol) and imidazole (2 mmol) under N_2 atmosphere using CH_2I_2 as a solvent. The reaction mixture was stirred for 12 h before concentrating and purifying through silica gel chromatography. Finally, The solution of compound **10b** (1 mmol) in CH_2Cl_2 (8 mL) and

pyridine (2 mL) was stirred at ice cold temperature for 15 minutes under N_2 atmosphere before the addition of chloroacetic anhydride (1.2 mmol). After 20 minutes, the mixture was washed with 1 N HCl and brine. The organic layer was collected, dried over Na_2SO_4, filtered, concentrated and purified through silica gel column chromatography to obtain compound **11** in three steps yield of 63 %. ^{1}H NMR (400 MHz, Chloroform-d) **(α anomer)** δ 7.87 – 7.83 (m, 3H), 7.78 (s, 1H), 7.64 – 7.61 (m, 4H), 7.51 – 7.48 (m, 2H), 7.43 – 7.30 (m, 9H), 7.06 (d, *J* = 7.9 Hz, 2H), 5.59 (d, *J* = 5.4 Hz, 1H), 5.28 – 5.23 (m, 1H), 5.07 (d, *J* = 11.6 Hz, 1H), 4.80 (d, *J* = 11.6 Hz, 1H), 4.37 (ddd, *J* = 10.1, 4.2, 2.3 Hz, 1H), 4.03 (dd, *J* = 10.2, 5.4 Hz, 1H), 3.86 – 3.81 (m, 1H), 3.67 (qd, *J* = 11.7, 3.3 Hz, 2H), 3.53 – 3.44 (m, 2H), 2.33 (s, 3H), 1.02 (s, 9H). ^{13}C NMR (101 MHz, Chloroform-d) δ 165.85, 138.06, 135.81, 135.67, 134.92, 134.89, 133.28, 133.12, 133.11, 132.96, 132.43, 130.01, 129.81, 129.72, 129.65, 128.47, 128.13, 127.79, 127.70, 127.17, 126.40, 126.31, 126.15, 87.46, 79.35, 75.46, 71.68, 71.56, 64.24, 62.35, 40.43, 26.77, 21.24, 19.28. **(β anomer)** ^{1}H NMR (400 MHz, Chloroform-*d*) δ 7.87-7.85 (m, 3H), 7.78 – 7.74 (m, 3H), 7.70 (dd, *J* = 7.7, 1.4 Hz, 2H), 7.57 (d, *J* = 8.1 Hz, 2H), 7.54 – 7.51 (m, 2H), 7.47 – 7.41 (m, 7H), 7.09 (d, *J* = 8.0 Hz, 2H), 5.21 (t, *J* = 6.8 Hz, 1H), 5.03 (d, *J* = 11.5 Hz, 1H), 4.80 (d, *J* = 11.6 Hz, 1H), 4.43 (d, *J* = 10.0 Hz, 1H), 3.80 (dd, *J* = 11.6, 1.9 Hz, 1H), 3.68 – 3.64 (m, 1H), 3.58 (dd, *J* = 13.2, 5.4 Hz, 1H), 3.51 – 3.45 (m, 4H), 2.36 (s, 3H), 1.10 (s, 9H).

Compound 13

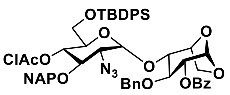

13

^{1}H NMR (400 MHz, Chloroform-d) δ 8.08 – 8.06 (m, 2H), 7.85 (td, *J* = 7.0, 6.4, 2.9 Hz, 3H), 7.78 (s, 1H), 7.64 (dt, *J* = 8.0, 1.4 Hz, 4H), 7.61 – 7.58 (m, 1H), 7.51 – 7.36 (m, 12H), 7.30 – 7.27 (m, 2H), 7.26 – 7.22 (m, 2H), 5.55 (d, *J* = 1.7 Hz, 1H), 5.36 (d, *J* = 3.8 Hz, 1H), 5.16 – 5.09 (m, 2H), 5.06 (d, *J* = 11.5 Hz, 1H), 4.91 (d, *J* = 10.8 Hz, 1H), 4.80 (dd, *J* = 11.0, 9.5 Hz, 2H), 4.65 (t, *J* = 4.5 Hz, 1H), 4.19 (d, *J* = 7.6 Hz, 1H), 4.12 (t, *J* = 8.0 Hz, 1H), 4.07 (dd, *J* = 8.1, 4.0 Hz, 1H), 4.03 – 3.98 (m, 1H), 3.77 – 3.72 (m, 2H), 3.67 (dd, *J* = 11.4, 5.3 Hz, 1H), 3.62 – 3.59 (m, 1H), 3.57 (d, *J* = 2.1 Hz, 2H), 3.51 (dd, *J* = 10.3, 3.8 Hz, 1H), 1.06 (s, 9H).

Compound 16

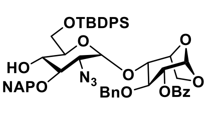

16

The solution of compound **13** (1 mmol) in MeOH (5 mL) and pyridine (5 mL) along with thiourea (1.4 mmol) was refluxed at 80 ^{o} C. After 1 h, volatiles were evaporated and residue was extracted with ethyl acetate, 1 N HCl/ brine and purified through silica gel column chromatography to obtain compound **16** in 92 % yield. ^{1}H NMR (400 MHz, Chloroform-d) δ 8.06 (dd, *J* = 8.3, 1.2 Hz, 2H), 7.86 (dq, *J* = 9.6, 4.2 Hz, 4H), 7.68 (td, *J* = 7.6, 7.0, 1.5 Hz, 4H), 7.62 – 7.55 (m, 2H), 7.53 – 7.38 (m, 11H), 7.26 – 7.20 (m, 4H), 5.54 (d, J = 1.7 Hz, 1H), 5.26 (d, *J* = 3.7 Hz, 1H), 5.12 (d, *J* = 11.3 Hz, 1H), 5.08 – 5.03 (m, 2H), 4.90 (d, *J* = 10.9 Hz, 1H), 4.76 (d, *J* = 10.9 Hz, 1H), 4.56 (t, *J* = 4.6 Hz, 1H), 4.17 (d, *J* = 7.7 Hz, 1H), 4.08 (t, *J* = 8.1 Hz, 1H), 4.00 (dd, *J* = 8.1, 4.1 Hz, 1H), 3.91 – 3.82 (m, 3H), 3.72 (t, *J* = 6.2 Hz, 2H), 3.61 (dt, *J* = 9.4, 4.5 Hz, 1H), 3.37 (dd, *J* = 10.3, 3.7 Hz, 1H), 2.66 (s, 1H), 1.08 (s, 9H).

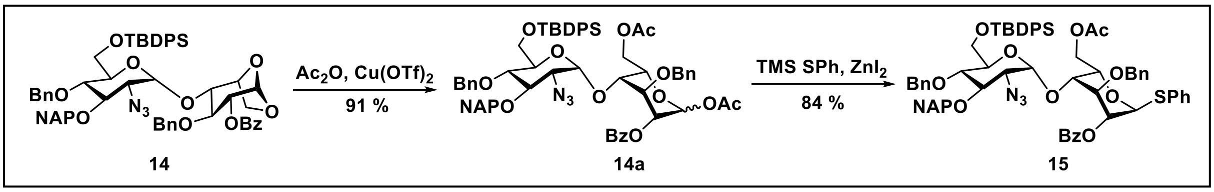

Compound 15

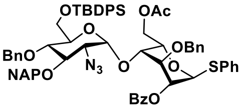

15

^{1}H NMR (400 MHz, Chloroform-d) δ 8.20 (dd, *J* = 6.8, 2.9 Hz, 2H), 7.87 – 7.77 (m, 3H), 7.72 (s, 1H), 7.71 – 7.68 (m, 2H), 7.68 – 7.66 (m, 1H), 7.61 – 7.58 (m, 3H), 7.54 – 7.48 (m, 4H), 7.44- 7.41 (m, 4H), 7.40 (s, 1H), 7.38 (d, *J* = 2.2 Hz, 4H), 7.37 – 7.35 (m, 4H), 7.34- 7.32 (m, 4H), 7.31- 7.29 (m, 2H), 7.21- 7.19 (m, 2H), 5.64 (s, 1H), 5.42 (s, 1H), 5.01 (d, *J* = 11.6 Hz, 1H), 4.92 (dt, *J* = 6.9, 4.0 Hz, 1H), 4.83 (d, *J* = 10.9 Hz, 1H), 4.78 (d, *J* = 11.7 Hz, 1H), 4.72 (d, *J* = 10.8 Hz, 1H), 4.65 (d, *J* = 3.6 Hz, 1H), 4.57 (d, *J* = 10.6 Hz, 1H), 4.44 (dd, *J* = 11.5, 8.1 Hz, 1H), 4.26 (d, *J* = 10.6 Hz, 1H), 4.20 (dd, *J* = 10.7, 3.6 Hz, 2H), 4.02 (dd, *J* = 11.6, 2.1 Hz, 1H), 3.91 (d, *J* = 11.5 Hz, 1H), 3.81 (s, 1H), 3.79 – 3.76 (m, 1H), 3.73 (d, *J* = 9.8 Hz, 1H), 3.68 (s, 1H), 3.38 (dd, *J* = 9.9, 3.6 Hz, 1H), 1.93 (s, 3H), 1.08 (s, 9H).

Compound 17

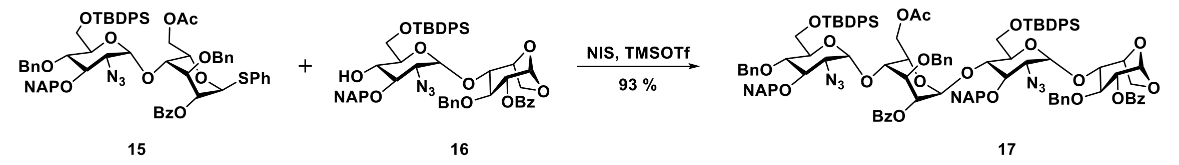

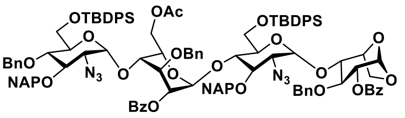

17

Yield 93 %. ^{1}H NMR (400 MHz, Chloroform-d) δ 8.03 (dd, *J* = 6.5, 1.4 Hz, 4H), 7.83 – 7.81 (m, 1H), 7.78 – 7.75 (m, 1H), 7.71- 7.58 (m, 15H), 7.48 – 7.27 (m, 29H), 7.22-7.19 (m, 9H), 5.51 (d, *J* = 1.7 Hz, 1H), 5.36 (s, 1H), 5.22 (s, 1H), 5.18 (d, J = 3.9 Hz, 1H), 5.13 (d, *J* = 11.3 Hz, 1H), 5.04 (dd, *J* = 8.2, 1.7 Hz, 1H), 4.87 (d, *J* = 11.3 Hz, 1H), 4.83 (d, *J* = 4.2 Hz, 1H), 4.81 – 4.80 (m, 1H), 4.75 (s, 1H), 4.73 – 4.67 (m, 3H), 4.63 (d, J = 10.6 Hz, 1H), 4.50 (dt, J = 6.8, 3.5 Hz, 1H), 4.41- 4.38 (m, 1H), 4.36 (d, J = 4.78 Hz, 1H), 4.18 – 4.06 (m, 4H), 4.01 (tt, *J* = 7.0, 3.9 Hz, 2H), 3.93 – 3.86 (m, 4H), 3.83 (dd, *J* = 9.4, 2.9 Hz, 2H), 3.74 (t, *J* = 9.8 Hz, 3H), 3.66 – 3.61 (m, 2H), 3.59 – 3.51 (m, 2H), 3.38 (dd, *J* = 10.4, 3.8 Hz, 1H), 3.31 (dd, *J* = 10.1, 3.5 Hz, 1H), 1.41 (s, 3H), 1.03 (s, 9H), 1.00 (s, 9H).

Compound 18

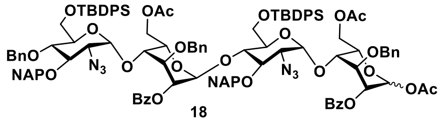

18

Yield 88%. ^{1}H NMR (400 MHz, Chloroform-d) δ 8.16 – 8.11 (m, 3H), 8.05 (d, *J* = 7.7 Hz, 3H), 7.86 – 7.77 (m, 4H), 7.64 (ddq, *J* = 22.1, 13.3, 7.6, 6.4 Hz, 21H), 7.56 – 7.29 (m, 50H), 7.28 – 7.14 (m, 17H), 6.24 (d, *J* = 2.3 Hz, 1H), 5.42 (s, 1H), 5.25 – 5.16 (m, 3H), 4.95 – 4.67 (m, 13H), 4.61 (dd, *J* = 7.2, 3.4 Hz, 2H), 4.41 (dq, *J* = 16.1, 6.1, 5.4 Hz, 5H), 4.30 – 4.10 (m, 8H), 4.09 – 4.01 (m, 3H), 3.93 – 3.63 (m, 18H), 3.52 (s, 1H), 3.35 – 3.30 (m, 3H), 2.12 (d, *J* = 3.9 Hz, 2H), 1.97 (s, 2H), 1.90 (s, 3H), 1.28 (s, 2H), 1.25 (s, 3H), 1.05 (s, 15H), 1.00 (s, 13H).

Compound 19

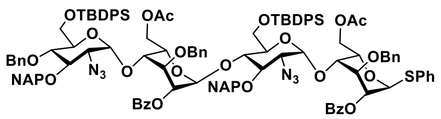

19

Yield 86 %. ^{1}H NMR (400 MHz, Chloroform-d) δ 8.12 (ddd, J = 5.7, 3.0, 1.6 Hz, 2H), 8.03 (s, 1H), 8.01 (d, J = 1.3 Hz, 1H), 7.82 – 7.79 (m, 1H), 7.77 – 7.74 (m, 1H), 7.67 – 7.56 (m, 15H), 7.48 – 7.43 (m, 7H), 7.40 – 7.37 (m, 7H), 7.35 – 7.33 (m, 4H), 7.31 – 7.28 (m, 14H), 7.25- 7.21 (m, 5H), 7.17 (d, *J* = 7.5 Hz, 2H), 7.14 – 7.10 (m, 4H), 5.62 (s, 1H), 5.38 (s, 1H), 5.34 (s, 1H), 5.18 (s, 1H), 4.94 – 4.83 (m, 3H), 4.79 (d, *J* = 10.8 Hz, 1H), 4.75 (d, *J* = 4.0 Hz, 1H), 4.72 (d, *J* = 4.1 Hz, 1H), 4.70 (d, *J* = 2.4 Hz, 1H), 4.67 (d, *J* = 3.2 Hz, 1H), 4.64 (d, *J* = 3.5 Hz, 1H), 4.56 (d, *J* = 10.6 Hz, 1H), 4.47 (d, J = 3.8 Hz, 1H), 4.38 (dd, *J* = 5.5, 3.2 Hz, 1H), 4.36 – 4.32 (m, 2H), 4.14 – 4.08 (m, 2H), 4.05 – 4.02 (m, 3H), 3.99 (t, *J* = 5.4 Hz, 1H), 3.91 (dd, *J* = 5.6, 1.9 Hz, 1H), 3.88 – 3.85 (m, 2H), 3.83 (t, *J* = 3.4 Hz, 1H), 3.79 (d, *J* = 10.2 Hz, 1H), 3.74 (d, *J* = 9.9 Hz, 1H), 3.71 – 3.66 (m, 2H), 3.63 – 3.57 (m, 2H), 3.54 – 3.51 (m, 1H), 3.47 (s, 1H), 3.28 (d, J = 3.5 Hz, 1H), 3.26 (d, J = 3.6 Hz, 1H), 1.86 (s, 3H), 1.20 (s, 3H), 1.02 (s, 9H), 0.97 (s, 9H

Compound 20

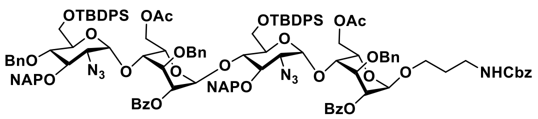

20

Yield 84 %. ^{1}H NMR (400 MHz, Chloroform-d) δ 8.09 (dd, J = 6.4, 3.1 Hz, 2H), 8.01 – 7.99 (m, 1H), 7.82 – 7.78 (m, 1H), 7.76 – 7.74 (m, 1H), 7.67 – 7.54 (m, 14H), 7.49 (s, 1H), 7.47 – 7.45 (m, 2H), 7.43 (s, 1H), 7.40 (s, 1H), 7.39 (s, 2H), 7.37 (s, 2H), 7.34 – 7.27 (m, 24H), 7.24 – 7.20 (m, 6H), 7.17 – 7.11 (m, 6H), 5.63 (s, 1H), 5.37 (s, 1H), 5.18 (s, 1H), 5.08 (s, 2H), 5.03 (s, 1H), 4.96 (s, 1H), 4.88 (dd, *J* = 11.4, 4.5 Hz, 1H), 4.78 (d, *J* = 10.7 Hz, 2H), 4.74 (d, *J* = 11.3 Hz, 2H), 4.68 (d, *J* = 10.8 Hz, 1H), 4.64 (d, *J* = 3.6 Hz, 1H), 4.58 (dd, *J* = 17.4, 11.3 Hz, 2H), 4.39 (dt, *J* = 8.6, 4.4 Hz, 1H), 4.33 (dt, *J* = 11.2, 3.9 Hz, 2H), 4.27 – 4.24 (m, 2H), 4.10 (t, *J* = 9.4 Hz, 2H), 4.03 (d, *J* = 12.5 Hz, 1H), 3.99 – 3.93 (m, 3H), 3.89 (s, 1H), 3.87 – 3.80 (m, 5H), 3.74 (d, J = 11.9 Hz, 1H), 3.68 (t, *J* = 9.7 Hz, 2H), 3.61 (d, *J* = 10.3 Hz, 2H), 3.57 – 3.54 (m, 3H), 3.47 (s, 1H), 3.39 (s, 1H), 3.28

(d, *J* = 3.6 Hz, 1H), 3.25 (d, *J* = 3.3 Hz, 1H), 3.24 – 3.18 (m, 2H), 1.86 (s, 3H), 1.18 (s, 3H), 1.01 (s, 9H), 0.97 (s, 9H).

Compound 21

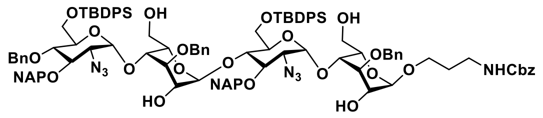

21

To a solution of compound **20** (0.84 g, 0.37 mmol) in CH_2Cl_2 (7 mL) and MeOH (7 mL) was added NaOMe (0.06g, 1.11 mmol) and stirred at room temperature. After 12 h, reaction mixture was quenched using Amberlite IR 120H^+ resin, filtered, evaporated and purified through silica gel column chromatography (ethyl acetate/ hexane= 1/2.5, v/v) to obtain compound **21** in 86 % yield. ^{1}H NMR (400 MHz, Chloroform-d) δ 7.81 – 7.68 (m, 13H), 7.57 (d, J = 8.5 Hz, 2H), 7.51 – 7.44 (m, 15H), 7.41 – 7.35 (m, 11H), 7.33 – 7.30 (m, 4H), 7.29 – 7.27 (m, 6H), 7.17 – 7.14 (m, 3H), 5.72 (s, 1H), 5.10 (s, 1H), 5.05 (s, 3H), 5.01 – 4.94 (m, 2H), 4.88 – 4.84 (m, 2H), 4.80 (dd, *J* = 10.6, 4.7 Hz, 2H), 4.73 (d, *J* = 11.3 Hz, 2H), 4.68 (dd, *J* = 10.9, 3.7 Hz, 2H), 4.63 – 4.51 (m, 2H), 4.25 (dt, *J* = 14.0, 6.9 Hz, 2H), 3.99 (t, *J* = 9.5 Hz, 1H), 3.89 – 3.83 (m, 7H), 3.81 – 3.71 (m, 8H), 3.61 – 3.54 (m, 8H), 3.48 (d, J = 11.5 Hz, 1H), 3.36 (d, *J* = 8.3 Hz, 1H), 3.22 (ddt, *J* = 18.0, 13.6, 5.6 Hz, 2H), 2.70 (dt, *J* = 10.7, 5.2 Hz, 1H), 2.16 (s, 1H), 1.85 (s, 2H), 1.10 (s, 9H), 1.05 (s, 9H).

Compound 22

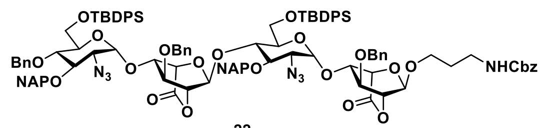

22

The solution of compound **21** (1 mmol) in CH_2Cl_2 / H_2O (1:1) along with TEMPO (0.2 mmol), BAIB (5, mmol) was stirred at room temperature for 16 h. After that the reaction mixture was extracted using saturated aqueous NH_4Cl. The collected organic layer was dried over Na_2SO_4, filtered, concentrated and purified through silica gel column chromatography **22** in 63 % yield. ^{1}H NMR (400 MHz, Chloroform-d) δ 7.89 – 7.78 (m, 7H), 7.71 – 7.68 (m, 4H), 7.65 – 7.61 (m, 4H), 7.51 – 7.29 (m, 36H), 7.22 – 7.17 (m, 3H), 5.84 (s, 1H), 5.47 (s, 1H), 5.25 (d, J = 12.0 Hz, 1H), 5.20 –

5.05 (m, 4H), 4.99 (d, J = 10.7 Hz, 1H), 4.95 (s, 1H), 4.92 (s, 1H), 4.87 (d, J = 11.6 Hz, 1H), 4.82 (d, *J* = 11.5 Hz, 1H), 4.76 (d, *J* = 8.9 Hz, 1H), 4.70 (d, *J* = 20.3 Hz, 1H), 4.65 – 4.62 (m, 1H), 4.49 (d, *J* = 12.4 Hz, 2H), 4.32 (d, *J* = 2.8 Hz, 1H), 4.24 (s, 1H), 4.21 – 4.14 (m, 3H), 4.01 – 3.85 (m, 5H), 3.82 – 3.76 (m, 3H), 3.71 (dt, *J* = 8.0, 3.3 Hz, 3H), 3.65 – 3.59 (m, 3H), 3.53 (dd, *J* = 14.1, 3.7 Hz, 1H), 3.48 – 3.45 (m, 2H), 3.34 (dt, *J* = 13.6, 6.6 Hz, 1H), 2.77 (dd, *J* = 9.4, 4.4 Hz, 1H), 1.89 – 1.87 (m, 2H), 1.07 (s, 18H).

Compound 23

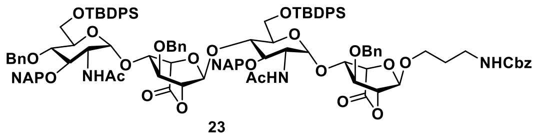

23

The solution of compound **22** (1 mmol) in THF/ AcOH/Ac_2O (3:2:2) was stirred along with Zn dust (40 mmol) for 12 h at room temperature. Upon completion, the reaction mixture was filtered through celite and volatiles were evaporated. The remaining residue was extracted with ethyl acetate, saturated $NaHCO_3$ and washed with brine and purified through silica gel column chromatography (ethyl acetate/ hexane= 1/2.5, v/v) to obtain compound **23** in 72 % yield. ^{1}H NMR (400 MHz, Chloroform-*d*) δ 7.92 – 7.86 (m, 3H), 7.82- 7.81 (m, 2H), 7.78 (d, *J* = 8.5 Hz, 1H), 7.76 – 7.74 (m, 1H), 7.71- 7.70 (m, 2H), 7.69 – 7.68 (m, 3H), 7.68 – 7.66 (m, 2H), 7.65 (d, *J* = 1.3 Hz, 1H), 7.58 (s, 1H), 7.53 (dt, *J* = 6.1, 2.7 Hz, 2H), 7.51 – 7.49 (m, 1H), 7.48 – 7.46 (m, 2H), 7.45 – 7.42 (m, 3H), 7.41 (s, 2H), 7.39 – 7.38 (m, 2H), 7.38 – 7.37 (m, 1H), 7.35- 7.31 (m, 7H), 7.29 (s, 1H), 7.28 – 7.27 (m, 7H), 7.26 – 7.24 (m, 3H), 7.23 – 7.20 (m, 3H), 7.18 – 7.16 (m, 2H), 7.05 (d, *J* = 7.3 Hz, 2H), 5.50 (s, 1H), 5.42 (t, *J* = 5.2 Hz, 1H), 5.16 (d, *J* = 3.6 Hz, 1H), 5.07 – 5.03 (m, 4H), 5.00 (s, 1H), 4.93 – 4.90 (m, 3H), 4.80 (d, *J* = 3.5 Hz, 1H), 4.77 (d, *J* = 2.1 Hz, 1H), 4.73 (d, *J* = 6.9 Hz, 1H), 4.63 (d, *J* = 12.4 Hz, 1H), 4.48 (s, 1H), 4.42 (d, *J* = 3.4 Hz, 1H), 4.37 (s, 1H), 4.33 (d, *J* = 2.5 Hz, 2H), 4.30 (d, *J* = 5.4 Hz, 1H), 4.27 – 4.21 (m, 1H), 4.09 (qd, *J* = 6.2, 4.2, 3.7 Hz, 3H), 4.02 – 3.89 (m, 7H), 3.86 – 3.70 (m, 2H), 3.68 – 3.59 (m, 5H), 3.53 (q, *J* = 10.8, 9.0 Hz, 2H), 3.36 (dt, *J* = 11.3, 6.0 Hz, 1H), 3.27 (dt, *J* = 13.8, 6.7 Hz, 1H), 1.83 – 1.80 (m, 2H), 1.59 (s, 3H), 1.39 (s, 3H), 1.09 (s, 9H), 1.06 (s, 9H).

Compound 24

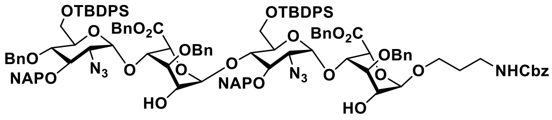

24

Yield 75 %. ^{1}H NMR (400 MHz, Chloroform-d) δ 7.74 – 7.66 (m, 3H), 7.65 – 7.41 (m, 13H), 7.38 – 7.35 (m, 2H), 7.33 – 7.11 (m, 37H), 7.07 (ddd, J = 13.9, 6.6, 2.9 Hz, 4H), 6.94 (ddt, J = 8.6, 3.3, 1.7 Hz, 1H), 6.85 (t, J = 7.5 Hz, 2H), 6.77 (d, J = 7.2 Hz, 2H), 5.44 – 5.36 (m, 2H), 5.04 (d, J = 12.4 Hz, 1H), 4.96 (d, J = 3.6 Hz, 1H), 4.93 – 4.88 (m, 4H), 4.77 – 4.73 (m, 4H), 4.70 – 4.67 (m, 2H), 4.61 (dd, J = 12.2, 6.2 Hz, 2H), 4.56 (d, J = 2.7 Hz, 2H), 4.53 (d, J = 4.4 Hz, 1H), 4.51 – 4.39 (m, 3H), 4.08 (t, J = 9.5 Hz, 1H), 3.95 – 3.93 (m, 2H), 3.85 (t, J = 3.9 Hz, 1H), 3.76 (ddd, J = 22.1, 12.9, 7.5 Hz, 5H), 3.68 (d, J = 4.5 Hz, 1H), 3.64 – 3.57 (m, 4H), 3.53 – 3.47 (m, 3H), 3.43 – 3.30 (m, 5H), 3.19 (d, J = 10.6 Hz, 1H), 3.16 – 3.11 (m, 1H), 1.78 – 1.65 (m, 2H), 0.98 (s, 9H), 0.94 (s, 9H).

Compound 25

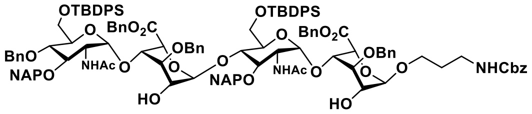

25

Yield 73 %. ^{1}H NMR (400 MHz, Chloroform-d) δ 7.82- 7.77 (m, 3H), 7.76 – 7.74 (m, 2H), 7.73 – 7.72 (m, 2H), 7.71 (d, J = 1.6 Hz, 1H), 7.70 (d, J = 1.6 Hz, 2H), 7.69 – 7.68 (m, 2H), 7.67 – 7.66 (m, 1H), 7.65 (d, J = 1.3 Hz, 1H), 7.63 (d, J = 1.4 Hz, 1H), 7.55 (s, 1H), 7.45 (dd, J = 6.2, 3.3 Hz, 2H), 7.42 (s, 1H), 7.41 – 7.40 (m, 2H), 7.39 (d, J = 1.5 Hz, 2H), 7.37 (d, J = 2.1 Hz, 2H), 7.36- 7.35 (m, 2H), 7.35 (s, 4H), 7.32 – 7.30 (m, 6H), 7.29 (s, 1H), 7.28 – 7.22 (m, 17H), 7.20- 7.16 (m, 5H), 6.95 (t, J = 7.7 Hz, 2H), 6.86 (d, J = 7.9 Hz, 2H), 5.77 – 5.75 (m, 1H), 5.45 (t, J = 5.3 Hz, 1H), 5.37 (s, 1H), 5.16 (s, 1H), 5.14 – 5.09 (m, 2H), 5.03 – 5.00 (m, 1H), 5.00 – 4.93 (m, 1H), 4.91 (s, 1H), 4.88 (s, 2H), 4.85 (s, 1H), 4.81 (s, 1H), 4.77 – 4.74 (m, 2H), 4.70 (dd, J = 11.7, 3.6 Hz, 2H), 4.62 (d, J = 12.3 Hz, 1H), 4.56 (d, J = 11.4 Hz, 1H),

4.48 (d, J = 5.4 Hz, 1H), 4.46 – 4.42 (m, 2H), 4.30 (td, J = 10.7, 3.0 Hz, 1H), 4.26 – 4.15 (m, 2H), 4.05 (s, 1H), 3.95 (d, J = 9.5 Hz, 1H), 3.92 – 3.84 (m, 3H), 3.84 – 3.81 (m, 1H), 3.78 (d, J = 12.1 Hz, 2H), 3.73 (dd, J = 5.9, 1.8 Hz, 1H), 3.71 – 3.65 (m, 3H), 3.65 – 3.62 (m, 3H), 3.58 – 3.53 (m, 2H), 3.53 – 3.50 (m, 1H), 3.39 (dd, J = 16.5, 7.9 Hz, 2H), 3.30 – 3.21 (m, 1H), 2.90 – 2.86 (m, 1H), 2.74 (s, 1H), 1.80 – 1.79 (m, 2H), 1.67 (s, 3H), 1.29 (s, 3H), 1.07 (s, 9H), 1.04 (s, 9H).

Compound 28

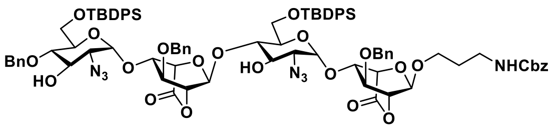

28

To a solution of compound **22** (1 mmol) in CH_2Cl_2 / H_2O (18 : 1) was added DDQ (5 mmol) portion wise over the interval of 20 min. After 1 h, the reaction mixture was quenched using $NaHCO_3$, extracted with CH_2Cl_2 and washed with brine. The collected organic layer was dried over Na_2SO_4, filtered, concentrated and purified through silica gel column chromatography (ethyl acetate/ hexane= 1/2.5, v/v) to obtain compound **28** in 53 % yield. ^1H NMR (400 MHz, Chloroform-d) δ 7.72 (t, J = 1.3 Hz, 1H), 7.70 – 7.67 (m, 3H), 7.63 – 7.61 (m, 4H), 7.46 – 7.42 (m, 3H), 7.41 – 7.34 (m, 12H), 7.33 – 7.29 (m, 13H), 7.26 – 7.23 (m, 4H), 5.82 (t, J = 5.4 Hz, 1H), 5.12 – 5.08 (m, 3H), 5.04 (dd, J = 2.3, 1.0 Hz, 1H), 4.99 (d, J = 3.8 Hz, 1H), 4.91 (d, J = 3.6 Hz, 1H), 4.81 – 4.69 (m, 3H), 4.62 (d, J = 12.4 Hz, 1H), 4.54 (d, J = 11.6 Hz, 1H), 4.45 – 4.42 (m, 2H), 4.36 (dd, J = 5.9, 3.3 Hz, 2H), 4.22 – 4.19 (m, 1H), 4.13 – 4.11 (m, 1H), 4.03 (t, J = 2.6 Hz, 1H), 4.00 – 3.92 (m, 5H), 3.90 (dd, J = 12.0, 1.8 Hz, 1H), 3.81 – 3.79 (m, 2H), 3.77 – 3.75 (m, 2H), 3.72 (s, 1H), 3.70 – 3.68 (m, 2H), 3.65 – 3.59 (m, 2H), 3.49 – 3.41 (m, 1H), 3.35 – 3.28 (m, 1H), 3.24 (dd, J = 10.4, 3.7 Hz, 1H), 3.18 (dd, J = 10.5, 3.7 Hz, 1H), 2.46 (s, 1H), 1.86 (s, 1H), 1.09 (s, 9H), 1.06 (s, 9H).

Compound 30

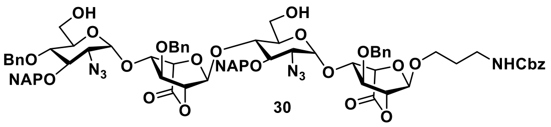

Yield 83 %. ^{1}H NMR (400 MHz, Chloroform-d) δ 7.92 (s, 1H), 7.89 – 7.76 (m, 6H), 7.72 (s, 1H), 7.53 – 7.50 (m, 2H), 7.49 – 7.45 (m, 3H), 7.44 – 7.40 (m, 2H), 7.38 (s, 2H), 7.37 – 7.31 (m 8H), 7.30 – 7.29 (m, 7H), 7.24 (dd, J = 7.5, 1.9 Hz, 2H), 5.75 (t, J = 5.1 Hz, 1H), 5.35 (s, 1H), 5.16 (d, J = 12.1 Hz, 1H), 5.09 – 5.07 (m, 3H), 5.01 (s, 1H), 4.98 – 4.93 (m, 3H), 4.79 – 4.70 (m, 3H), 4.67 – 4.64 (m, 1H), 4.62 – 4.56 (m, 2H), 4.52 (t, J = 3.4 Hz, 2H), 4.46 (t, J = 2.6 Hz, 1H), 4.30 – 4.28 (m, 2H), 3.95 – 3.85 (m, 5H), 3.82 – 3.76 (m, 2H), 3.73 (td, J = 3.1, 1.5 Hz, 1H), 3.71 – 3.68 (m, 3H), 3.66 – 3.61 (m, 2H), 3.57 (d, J = 3.8 Hz, 1H), 3.51 – 3.27 (m, 6H), 2.83 (dd, J = 10.4, 3.7 Hz, 1H), 2.05 (s, 1H), 1.85 (s, 2H).

Compound 31

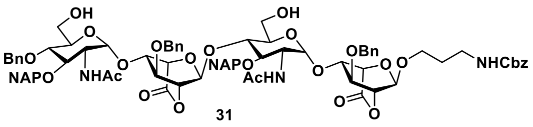

Yield 87 %. ^{1}H NMR (400 MHz, Chloroform-*d*) δ 7.87 (s, 1H), 7.84 (d, *J* = 10.4 Hz, 5H), 7.77 (s, 1H), 7.67 (s, 1H), 7.51 (dd, *J* = 7.8, 4.9 Hz, 4H), 7.45 (dd, *J* = 8.4, 1.5 Hz, 1H), 7.36 – 7.30 (m, 14H), 7.24 (d, *J* = 8.4 Hz, 3H), 7.20 – 7.17 (m, 2H), 7.08 (d, *J* = 6.7 Hz, 2H), 5.50 (t, *J* = 5.1 Hz, 1H), 5.41 (d, *J* = 0.8 Hz, 1H), 5.11 (d, *J* = 3.7 Hz, 1H), 5.08 – 5.03 (m, 3H), 5.01- 4.88 (m, 4H), 4.85 (d, *J* = 11.0 Hz, 1H), 4.75 – 4.67 (m, 4H), 4.59 (s, 1H), 4.53 – 4.52 (m, 1H), 4.47 (d, *J* = 2.8 Hz, 1H), 4.42 – 4.31 (m, 5H), 4.18 (d, *J* = 3.4 Hz, 1H), 4.13 – 4.07 (m, 2H), 3.95 – 3.87 (m, 3H), 3.78 (dd, *J* = 25.8, 10.9 Hz, 3H), 3.68 – 3.53 (m, 11H), 3.44 – 3.36 (m, 1H), 3.23 (ddd, *J* = 10.4, 9.5, 5.3 Hz, 1H), 1.81 (s, 2H), 1.52 (d, *J* = 9.8 Hz, 6H).

Compound 26

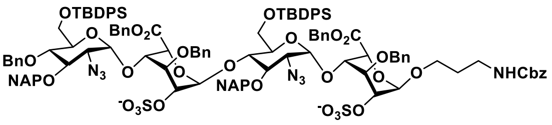

26

Yield 63 %. ^{1}H NMR (400 MHz, Methanol-d4) δ 7.79 – 7.76 (m, 7H), 7.74 – 7.70 (m, 3H), 7.61 – 7.41 (m, 13H), 7.38 – 7.30 (m, 14H), 7.29 – 7.15 (m, 23H), 7.04 (t, J = 6.6 Hz, 2H), 6.96 (t, J = 7.5 Hz, 2H), 6.65 (d, J = 7.7 Hz, 1H), 5.71 (s, 1H), 5.37 (d, J = 12.1 Hz, 1H), 5.22 (d, J = 3.5 Hz, 1H), 5.17 (s, 2H), 5.06 (s, 1H), 5.02 – 4.89 (m, 7H), 4.83 (d, J = 5.6 Hz, 1H), 4.77 – 4.69 (m, 4H), 4.63 – 4.51 (m, 3H), 4.48 (s, 1H), 4.38 (d, J = 11.7 Hz, 1H), 4.33 – 4.26 (m, 2H), 4.22 – 4.19 (m, 2H), 4.04 – 3.98 (m, 3H), 3.86 (d, J = 10.4 Hz, 2H), 3.82 – 3.70 (m, 4H), 3.65 – 3.54 (m, 4H), 3.29 – 3.12 (m, 3H), 1.81 – 1.73 (m, 2H), 0.99 (d, J = 6.8 Hz, 18H).

Compound 27

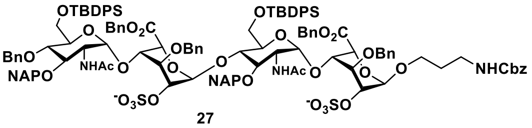

27

Yield 51 %. ^{1}H NMR (400 MHz, Methanol-d4) δ 7.83 (s, 1H), 7.82 – 7.80 (m, 2H), 7.79 (s, 2H), 7.76 – 7.73 (m, 3H), 7.71 (s, 1H), 7.68 (d, J = 7.6 Hz, 3H), 7.63 (s, 1H), 7.61 (d, J = 5.1 Hz, 2H), 7.58 (d, J = 6.1 Hz, 3H), 7.48 (dd, J = 16.8, 8.0 Hz, 4H), 7.43- 7.38 (m, 9H), 7.36- 7.30 (m, 16H), 7.25 – 7.20 (m, 7H), 7.19- 7.15 (m, 7H), 6.98 (d, J = 7.4 Hz, 2H), 5.71 (s, 1H), 5.20 (s, 4H), 4.98 (s, 3H), 4.83 (s, 1H), 4.81 – 4.75 (m, 3H), 4.69 (d, J = 12.0 Hz, 2H), 4.57 – 4.50 (m, 5H), 4.41 – 4.34 (m, 5H), 4.24 – 4.03 (m, 5H), 3.90 (d, J = 15.9 Hz, 2H), 3.81- 3.74 (m, 6H), 3.65 – 3.56 (m, 5H), 3.29 – 3.25 (m, 1H), 3.20- 3.13 (m, 1H), 2.01 (s, 3H), 1.84 – 1.75 (m, 5H), 1.07 (s, 9H), 0.98 (s, 9H).

Compound 29

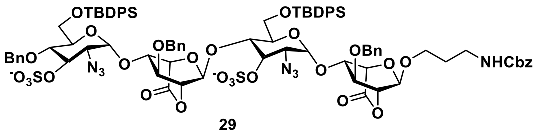
29

Yield 52 %. ^{1}H NMR (400 MHz, Methanol-d4) δ 7.68 – 7.67 (m, 1H), 7.66 (d, J = 2.0 Hz, 1H), 7.65 – 7.61 (m, 4H), 7.60 (s, 1H), 7.58 (d, J = 1.3 Hz, 1H), 7.41 (d, J = 7.4 Hz, 2H), 7.39 – 7.34 (m, 4H), 7.32 – 7.29 (m, 9H), 7.29 – 7.22 (m, 13H), 7.20 – 7.13 (m, 4H), 5.49 (s, 1H), 5.18 (d, J = 10.6 Hz, 1H), 5.13 (d, J = 3.4 Hz, 1H), 5.06 (s, 1H), 5.02- 5.00 (m, 2H), 4.98 – 4.87 (m, 2H), 4.76 – 4.70 (m, 5H), 4.56 (dd, J = 18.2, 11.0 Hz, 2H), 4.43 – 4.42 (m, 1H), 4.41- 4.39 (m, 2H), 4.35 – 4.32 (m, 1H), 4.07 – 3.94 (m, 3H), 3.87- 3.83 (m, 3H), 3.80- 3.65 (m, 5H), 3.55 (dt, J = 9.3, 5.5 Hz, 1H), 3.26 – 3.16 (m, 3H), 1.78 – 1.74 (m, 2H), 0.99 (s, 9H), 0.96 (s, 9H).

Compound 32

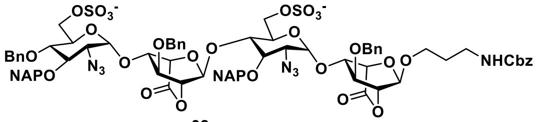
32

Yield 70 %. ^{1}H NMR (400 MHz, Methanol-d_4) δ 7.85 – 7.80 (m, 4H), 7.79 (d, *J* = 2.2 Hz, 1H), 7.76 – 7.74 (m, 2H), 7.70 (d, *J* = 8.4 Hz, 2H), 7.58- 7.54 (m, 2H), 7.52 – 7.50 (m, 1H), 7.49 (d, *J* = 2.1 Hz, 1H), 7.48 – 7.39 (m, 8H), 7.38 – 7.26 (m, 13H), 5.29 (s, 1H), 5.18 (d, *J* = 3.2 Hz, 1H), 5.15 (d, *J* = 3.5 Hz, 1H), 5.07 (s, 2H), 4.99 (d, *J* = 2.9 Hz, 1H), 4.93 (s, 2H), 4.82 (d, *J* = 3.1 Hz, 2H), 4.80 – 4.79 (m, 2H), 4.76- 4.75 (m, 5H), 4.34 – 4.26 (m, 2H), 4.23 (dt, *J* = 4.9, 2.4 Hz, 1H), 4.20 – 4.18 (m, 1H), 4.12 (d, *J* = 9.4 Hz, 1H), 4.04 (t, *J* = 3.8 Hz, 1H), 3.98- 3.93 (m, 3H), 3.91 – 3.84 (m, 3H), 3.78 – 3.68 (m, 4H), 3.67 – 3.58 (m, 3H), 3.55 (dd, *J* = 9.9, 3.5 Hz, 1H), 3.45 (d, *J* = 9.5 Hz, 1H), 3.26 (tt, *J* = 13.0, 6.5 Hz, 2H), 1.86- 1.79 (m, 2H).

Compound 33

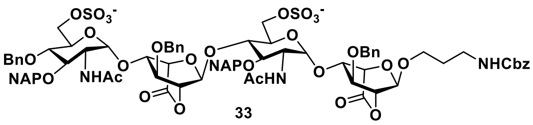

Yield 65 %. ^{1}H NMR (400 MHz, Methanol-d_4) δ 7.91 – 7.70 (m, 9H), 7.51 – 7.37 (m, 14H), 7.33 – 7.22 (m, 11H), 5.54 (s, 1H), 5.13 (d, *J* = 7.0 Hz, 2H), 5.09 (s, 1H), 5.06 (s, 2H), 4.81 – 4.52 (m, 9H), 4.46 (d, *J* = 8.9 Hz, 1H), 4.39 (d, *J* = 8.3 Hz, 2H), 4.32 – 4.27 (m, 3H), 4.25 – 4.16 (m, 3H), 4.10 – 3.89 (m, 8H), 3.83 – 3.54 (m, 6H), 3.26 – 3.15 (m, 2H), 1.84 – 1.75 (m, 8H).

Compound 34

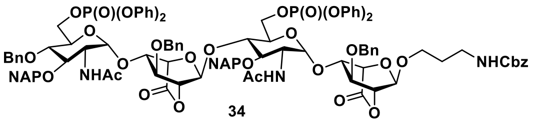

To a solution of compound **18** (1 mmol) in CH_2Cl_2 pyridine (1:1) was added NEt_3 (10 mmol), DMAP (0.3 mmol) and Diphenyl phosphoryl chloride (5 mmol) at 0 ^{o} C. After 12 h, the reaction mixture was diluted with CH_2Cl_2 and extracted with 1 N HCl / brine and purified through silica gel column chromatography (ethyl acetate/ hexane= 0.6/1, v/v) to obtain compound **34** in 60 % yield. ^{1}H NMR (400 MHz, Chloroform-*d*) δ 7.84 (dd, *J* = 12.9, 8.5 Hz, 6H), 7.73 (d, *J* = 20.7 Hz, 2H), 7.54 – 7.46 (m, 4H), 7.44 – 7.31 (m, 16H), 7.28 – 7.21 (m, 19H), 7.19 – 7.08 (m, 7H), 5.35 – 5.29 (m, 2H), 5.05 (s, 3H), 5.01 – 4.97 (m, 3H), 4.89 (d, *J* = 11.6 Hz, 2H), 4.78 – 4.69 (m, 3H), 4.63 (t, *J* = 2.6 Hz, 1H), 4.56 (dd, *J* = 15.6, 7.7 Hz, 3H), 4.52 – 4.42 (m, 4H), 4.41 – 4.33 (m, 4H), 4.31 – 4.27 (m, 2H), 4.21 (dd, *J* = 4.9, 3.3 Hz, 1H), 4.11 – 4.07 (m, 1H), 4.06 – 3.88 (m, 3H), 3.76 – 3.69 (m, 2H), 3.66 – 3.52 (m, 7H), 3.39 – 3.30 (m, 1H), 3.23 (dt, *J* = 12.9, 5.5 Hz, 1H), 1.81 (s, 2H), 1.51 (s, 3H), 1.42 (s, 3H).

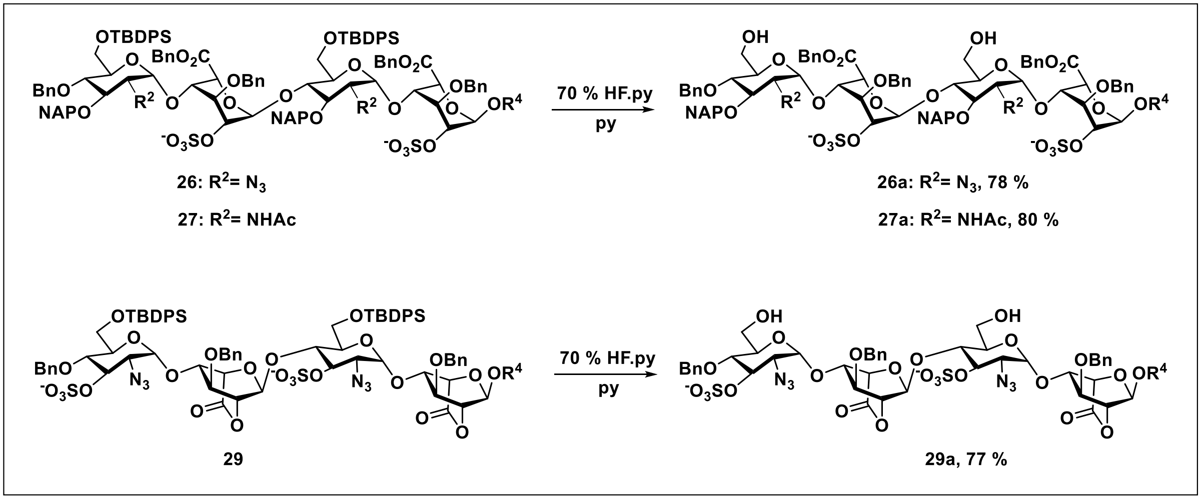

Compound 26a

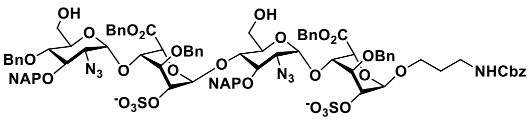

26a

^{1}H NMR (400 MHz, Methanol-d_4) δ 7.82 – 7.78 (m, 1H), 7.76- 7.74 (m, 2H), 7.72- 7.70 (m, 2H), 7.63 (dd, J = 16.5, 8.2 Hz, 2H), 7.47- 7.39 (m, 10H), 7.36- 7.34 (m, 5H), 7.32- 7.27 (m, 10H), 7.26 – 7.24 (m, 5H), 7.17 – 7.10 (m, 3H), 7.02 (t, J = 7.5 Hz, 2H), 6.62 (d, J = 7.5 Hz, 2H), 5.51 (s, 1H), 5.41 (d, J = 12.1 Hz, 1H), 5.27 – 5.26 (m, 1H), 5.20 (s, 1H), 5.15 (d, J = 3.0 Hz, 1H), 5.09 – 5.06 (m, 2H), 5.02 – 4.97 (m, 4H), 4.94 – 4.89 (m, 2H), 4.79 – 4.69 (m, 3H), 4.64 – 4.62 (m, 1H), 4.62 – 4.50 (m, 4H), 4.39- 4.35 (m, 3H), 4.18 – 4.14 (m, 1H), 4.12 – 4.03 (m, 2H), 3.99 – 3.73 (m, 7H), 3.61- 3.51 (m, 3H), 3.48 – 3.38 (m, 3H), 3.31 – 3.13 (m, 4H), 1.84- 1.76 (m, 2H).

Compound 27a

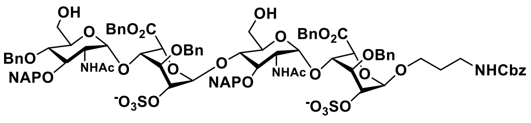

27a

^{1}H NMR (400 MHz, Methanol-d_4) δ 7.68 – 7.65 (m, 2H), 7.63- 7.60 (m, 3H), 7.53 – 7.45 (m, 4H), 7.34 (d, J = 3.3 Hz, 2H), 7.32 – 7.31 (m, 4H), 7.28- 7.25 (m, 7H), 7.20- 7.18 (m, 7H), 7.16 –

7.12 (m, 5H), 7.08 (dd, J = 5.0, 1.9 Hz, 3H), 7.05 (dd, J = 8.5, 1.5 Hz, 2H), 6.99 (dd, J = 6.6, 3.1 Hz, 2H), 6.95 (d, J = 7.4 Hz, 1H), 6.81 (t, J = 7.7 Hz, 2H), 5.43 (s, 1H), 5.21 (d, J = 12.0 Hz, 1H), 5.07 (d, J = 9.6 Hz, 2H), 4.97- 4.94 (m, 2H), 4.87 (d, J = 2.9 Hz, 1H), 4.68 – 4.66 (m, 2H), 4.61 (dd, J = 11.1, 4.7 Hz, 3H), 4.48 (d, J = 5.3 Hz, 3H), 4.44 (dd, J = 9.1, 3.0 Hz, 3H), 4.39 (d, J = 9.3 Hz, 2H), 4.16 (s, 3H), 4.04 – 3.98 (m, 4H), 3.85 (t, J = 12.0 Hz, 4H), 3.67- 3.60 (m, 4H), 3.55 – 3.41 (m, 5H), 3.30- 3.26 (m, 2H), 3.11 – 3.05 (m, 2H), 1.93 (s, 3H), 1.74 – 1.68 (m, 2H), 1.61 (s, 3H).

Compound 29a

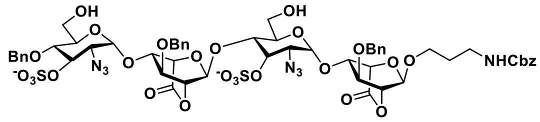

29a

1H NMR (400 MHz, Methanol-d4) δ 7.50 – 7.41 (m, 7H), 7.38- 7.37 (m, 3H), 7.34 – 7.25 (m, 10H), 5.40 (dd, J = 7.5, 2.8 Hz, 2H), 5.24 – 5.13 (m, 4H), 5.07 (s, 2H), 4.94 (dd, J = 5.1, 3.0 Hz, 1H), 4.83 – 4.78 (m, 3H), 4.76 (d, J = 3.7 Hz, 4H), 4.61 (d, J = 3.0 Hz, 1H), 4.57 (d, J = 10.5 Hz, 1H), 4.50 (d, J = 3.2 Hz, 1H), 4.41 – 4.37 (m, 1H), 3.95 – 3.88 (m, 3H), 3.88 – 3.83 (m, 4H), 3.83 – 3.57 (m, 2H), 3.26 (dt, J = 12.8, 6.1 Hz, 2H), 1.82 – 1.79 (m, 2H).

Compound 1

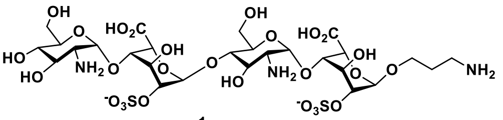

1

^{1}H NMR (600 MHz, Deuterium Oxide) δ 5.33 (s, 1H), 5.15 (s, 1H), 5.05 (s, 1H), 4.84 (s, 1H), 4.46 (s, 1H), 4.28 – 4.25 (m, 3H), 4.17 (s, 1H), 4.06 (d, J = 14.2 Hz, 2H), 3.88 (t, J = 9.9 Hz, 1H), 3.81 – 3.77 (m, 5H), 3.73 (s, 2H), 3.68- 3.67 (m, 2H), 3.64 – 3.62 (m, 1H), 3.40 (t, J = 9.6 Hz, 1H), 3.29 (d, J = 10.7 Hz, 1H), 3.26 – 3.24 (m, 1H), 3.09- 3.05 (m, 2H), 1.96- 1.87 (m, 2H).

Compound 2

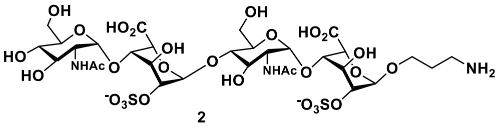

2

^{1}H NMR (600 MHz, Deuterium Oxide) δ 5.12 (s, 2H), 5.06 (s, 1H), 5.03 (d, *J* = 3.4 Hz, 1H), 5.01 (d, *J* = 3.4 Hz, 1H), 4.76 (d, *J* = 1.8 Hz, 1H), 4.21 (d, *J* = 6.5 Hz, 2H), 4.17 (d, *J* = 13.4 Hz, 2H), 3.97 (d, *J* = 11.1 Hz, 2H), 3.90 (ddd, *J* = 18.5, 10.5, 3.4 Hz, 2H), 3.85 – 3.81 (m, 1H), 3.76 (s, 2H), 3.71 (d, *J* = 2.8 Hz, 2H), 3.65 – 3.59 (m, 4H), 3.56 – 3.50 (m, 2H), 3.42 (d, *J* = 9.2 Hz, 1H), 3.06 (t, *J* = 6.3 Hz, 2H), 1.98 (s, 3H), 1.96 (s, 3H), 1.93 – 1.88 (m, 2H).

Compound 3

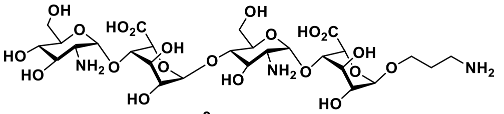

3

^{1}H NMR (400 MHz, Deuterium Oxide) δ 5.37 (d, J = 3.6 Hz, 1H), 5.29 (d, J = 3.7 Hz, 1H), 4.72 (d, J = 4.2 Hz, 1H), 4.59 (d, J = 3.2 Hz, 1H), 4.36 (d, J = 2.7 Hz, 1H), 4.03 – 4.01 (m, 1H), 3.96 (t, J = 3.1 Hz, 1H), 3.91 – 3.88 (m, 1H), 3.79 (dd, J = 7.0, 3.8 Hz, 1H), 3.76- 3.72 (m, 2H), 3.70 (s, 3H), 3.68- 3.66 (m, 3H), 3.65 – 3.63 (m, 1H), 3.61 (d, J = 9.4 Hz, 1H), 3.57- 3.56 (m, 1H), 3.55- 3.51 (m, 3H), 3.32 (t, J = 9.4 Hz, 1H), 3.19 (ddd, J = 12.6, 10.6, 3.6 Hz, 2H), 2.97 (t, J = 6.7 Hz, 2H), 1.87- 1.78 (m, 2H).

Compound 4

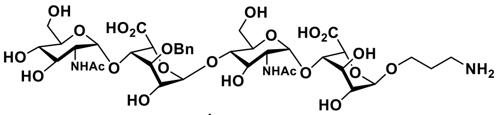

4

^{1}H NMR (400 MHz, Deuterium Oxide) δ 5.08 (d, J = 3.7 Hz, 1H), 5.05 (d, J = 3.8 Hz, 1H), 5.00 (d, J = 2.7 Hz, 1H), 4.90 (d, J = 2.7 Hz, 1H), 4.84 (d, J = 1.8 Hz, 1H), 4.67 (d, J = 2.4 Hz, 1H), 4.02 (t, J = 3.1 Hz, 1H), 3.99 (t, J = 2.6 Hz, 1H), 3.94 (t, J = 3.6 Hz, 1H), 3.91 – 3.79 (m, 4H),

3.75- 3.71 (m, 4H), 3.67 – 3.65 (m, 3H), 3.64 – 3.58 (m, 3H), 3.58 – 3.52 (m, 2H), 3.45 (d, J = 9.3 Hz, 1H), 3.06 (t, J = 6.6 Hz, 2H), 1.95 – 1.88 (m, 8H).

Compound 5

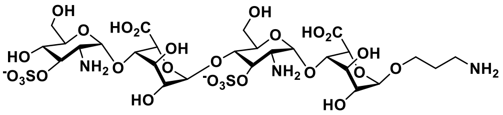

5

^{1}H NMR (600 MHz, Deuterium Oxide) δ 5.51 (d, J = 3.5 Hz, 1H), 5.41 – 5.39 (m, 1H), 4.95 (d, J = 2.8 Hz, 1H), 4.87 (d, J = 4.0 Hz, 1H), 4.56 – 4.50 (m, 1H), 4.48 – 4.42 (m, 2H), 4.19 (t, J = 3.6 Hz, 1H), 4.08 – 4.05 (m, 1H), 3.99 (t, J = 3.8 Hz, 1H), 3.93 (t, J = 9.6 Hz, 1H), 3.89 (s, 1H), 3.88 – 3.84 (m, 2H), 3.82 – 3.77 (m, 6H), 3.69- 3.67 (m, 1H), 3.64 – 3.59 (m, 4H), 3.55 (dd, J = 10.4, 3.6 Hz, 1H), 3.06 – 3.04 (m, 2H), 1.96 – 1.87 (m, 2H).

Compound 6

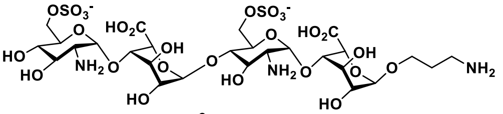

6

^{1}H NMR (600 MHz, Deuterium Oxide) δ 5.41 (d, J = 3.7 Hz, 1H), 5.39 (d, J = 3.6 Hz, 1H), 5.04 – 5.03 (m, 3H), 4.15 – 4.11 (m, 1H), 4.07 – 3.97 (m, 1H), 3.85 – 3.81 (m, 2H), 3.80- 3.72 (m, 5H), 3.70 – 3.59 (m, 5H), 3.50 – 3.39 (m, 2H), 3.30 (td, J = 10.7, 10.0, 3.6 Hz, 1H), 3.08- 3.05 (m, 2H), 2.76 (d, J = 5.2 Hz, 1H), 1.96- 1.88 (m, 2H).

Compound 7

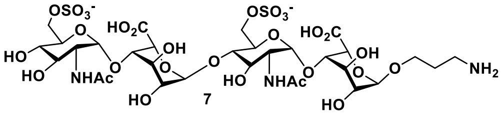

7

^{1}H NMR (400 MHz, Deuterium Oxide) δ 4.99 (t, *J* = 4.2 Hz, 2H), 4.95 (d, *J* = 2.4 Hz, 1H), 4.87 (s, 1H), 4.77 (s, 1H), 4.60 (d, *J* = 1.9 Hz, 1H), 4.16 (ddd, *J* = 11.0, 8.0, 3.3 Hz, 2H), 4.06 (ddd, *J* = 10.9, 6.7, 1.8 Hz, 2H), 3.94 (dt, *J* = 4.8, 2.8

Hz, 2H), 3.89 – 3.72 (m, 6H), 3.64- 3.62 (m, 2H), 3.60 – 3.53 (m, 4H), 3.50 (d, *J* = 10.4 Hz, 1H), 3.42 (d, *J* = 9.7 Hz, 1H), 2.99 (t, *J* = 6.6 Hz, 2H), 1.88- 1.80 (m, 8H).

Compound 8

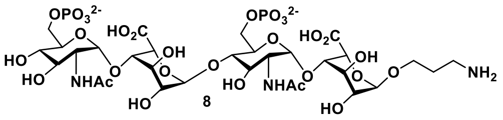

^{1}H NMR (400 MHz, Deuterium Oxide) δ 4.98 (d, *J* = 3.1 Hz, 2H), 4.89 (s, 1H), 4.78 (s, 1H), 3.99- 3.70 (m, 6H), 3.85 (ddd, *J* = 14.1, 8.1, 3.2 Hz, 4H), 3.80 – 3.73 (m, 1H), 3.69 – 3.49 (m, 9H), 3.48 – 3.40 (m, 1H), 2.99 (t, *J* = 6.4 Hz, 2H), 1.92- 1.82 (m, 8H).

4. Synthesis and characterization of Texas red conjugated Heparin (T-HP)

To a solution of commercially available Heparin sodium salt (5 mg/ mL) (H4784 Sigma) in 0.1 M sodium bicarbonate buffer (pH= 8.3) was added 100 µL of Texas Red™-X, Succinimidyl Ester (0.5 mg) in a drop wise manner and left for stirring for 1 h at room temperature. After 1 h, the solution was dialyzed against 1 kD dialysis membrane for 24 h to remove the unreacted dye solution. Finally, the dialysed solution was lyophilized and stored at -20 ^{o}C till further use.

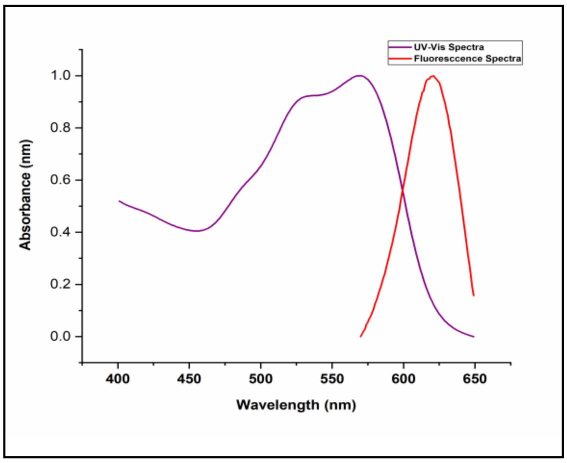

Figure S1: UV-Visible and Fluorescence Spectra

5. ELISA Protocol

IC_{50} value of heparinoids **7** & **8** with HB-EGF were evaluated using standard competitive ELISA protocol. First, commercially available Heparin sodium salt in PBS at a concentration of 10 µg/mL was added on 96 well flat bottomed (Nunc MaxiSorp) plate with 100 µL in coating buffer and incubated at RT for 16 h. For positive control HB-EGF at a concentration of 0.1 µg/mL was also coated and incubated. Next day, after blocking (at 37 ^oC) , pre-incubated mixture of HB-EGF (0.1 µg/mL) with an increasing heparinoid concentration (0- 1 mg/L, 100 µL) were added and incubated for 2 h at RT. After 2 h, normal washing procedure was followed and wells were incubated with biotinylated anti HB-EGF antibody (1 µg/mL, 100 µL) at RT for another 2 h. Next, plate was washed 3-4 times and incubated with streptavidin-HRP conjugate (0.05 µg/mL, 100 µL) for 30 minutes. Finally, TMB liquid substrate (100 µL) was added to wells after washing and incubated in dark at RT for 20 minutes for the color development. The reaction was stopped using 1 N HCl and absorbance was recorded at 450 nm and analysed using GraphPad Prism Software. A similar experiment with EGF and amphiepiregulin didn't showed any competitive binding in the presence of heparinoids concentration (0- 1 mg/L, 100 µL), indicating weak/no-binding affinity.

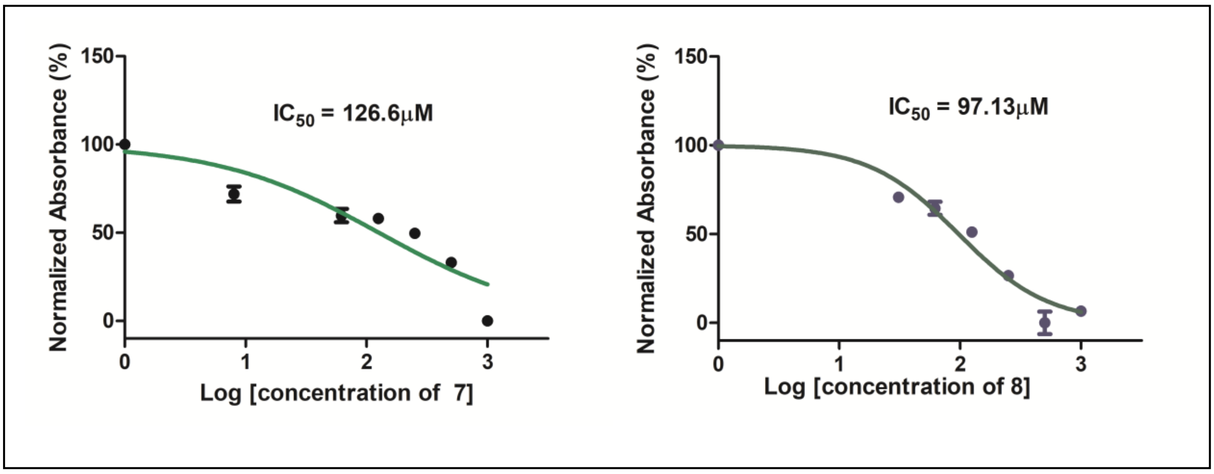

Figure S2: HB-EGF binding to heparin coated plate measured by competitive ELISA with heparinoid **7** and **8.**

6. SPR Analysis

Heparin tetrasaccharide ligands (**7** and **8**) were immobilized onto a CM5 chip (BIACORE) using standard amine coupling procedures. Briefly, the CM5 chip was activated by injecting a mixture of NHS/EDC mixture with a contact time of 300 S at a flow rate of 20 μL/min, followed by multiple injections of HS ligands (0.5 mM) dissolved in HBS-EP buffer. On the control channel, a propanol amine linker was injected. Different HB-EGF growth factors at a flow rate of 50 μL/min and 25 °C in HBS-EP buffer were injected for 250 secs. After that, dissociation was performed by injected the HBS-EP without growth factors. The binding was measured after reference subtraction of control. All data evaluation was performed using BIAevaluation.

**HS Tetrasaccharide**	**K_D (μM)**	**K_{on} ($M^{-1}S^{-1}$)**	**K_{off} (S^{-1})**
**7**	11.12 ± 0.3	$4.26 \pm 0.2 \times 10^4$	$4.75 \pm 0.32 \times 10^{-1}$
**8**	11.06 ± 0.15	$4.29 \pm 0.11 \times 10^4$	$4.72 \pm 0.22 \times 10^{-1}$

Table S1: SPR analysis of kinetic rate constants and equilibrium affinities for HS tetrasaccharides **7** and **8** binding to HB-EGF.

7. Synthesis of heparinoid-gold nanoparticles (AF_{555}Au@1 and AF_{555}Au@2).

The NHS activated-$Alexa_{555}$ fluorescent gold nanparticles (**AF_{555}Au**) were purchased from NanoPartz. The AF_{555}Au were suspended in PBS buffer in 1.5 mL centrifuge tube and incubated with **7** and **8** (3 mg) at 25 ºC for 12 h. After amide functionalization, the remaining NHS groups were neutralized with ethanolamine (0.05 μL) for 12 h. Subsequently, the nanoparticles were centrifuge at 15000 rpm for 5 minutes to precipitate functionalized

AuNPs. Next, nanoparticles were washed with PBS (3 X 1 ml) to remove unreacted heparinoids, ethanolamine and NHS. Finally, heparinoid-AuNPs were dissolved in PBS buffer and stored at 4 ºC till further use.

8. TEM, UV and Fluorescence measurements

TEM measurements of heparinoid- AuNPs were performed on a Jeol, JEM 2200FS. The samples were prepared by vacuum drying of 5 µl of the sample drop casted on carbon type-B 200 mesh copper grid.

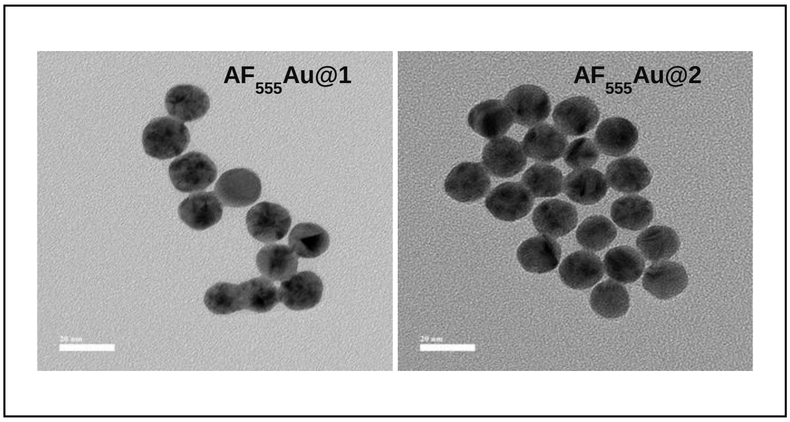

Figure S3: TEM Images (scale bar: 20 nm)

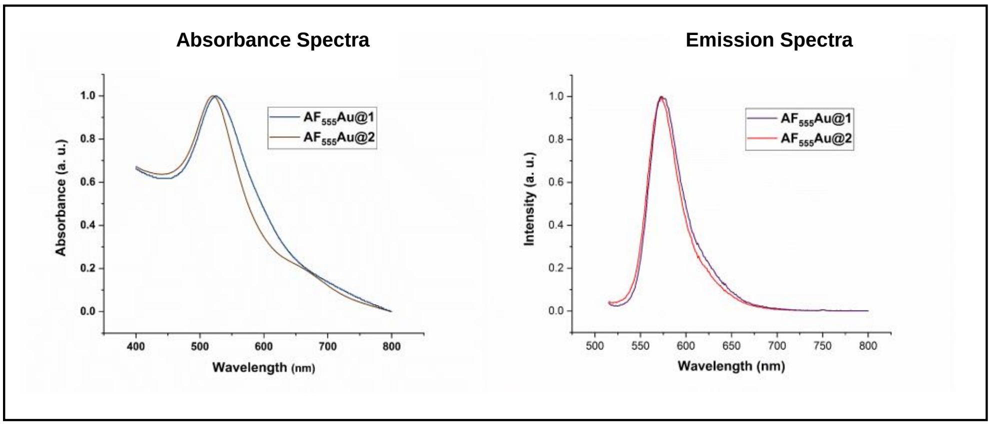

Figure S4: UV-Visible and Fluorescence Spectra.

Properties	Before Conjugation $AF_{555}Au$	After Conjugation $AF_{555}Au$	
		Au@1	Au@2
Size (nm)	10	15	15
λ_{max}(nm)	521	526	521
E_{max} (nm)	580	573	573
ζ (mV)	-20	-56.3	-59.4

Table S2: Physical characterization of $AF_{555}Au$.

9. Zeta potential measurements

Zeta potential was used to measure the electrophoretic mobility of heparinoid nanoparticles. We applied a unit field of 1 volt per meter to the nanoparticle solution and employed Helmholtz-Smoluchowski equation to quantify the zeta potentials.

10. Cell Viability assay.

MDA-MB-468 and NIH-3T3 (1×10^5 cells/well) were seeded in 96-well microtiter plate and incubated overnight in a 5% CO_2 incubator at 37 °C for attachment. Cells were then treated with heperinoid-AuNPS in different concentrations (0, 0.5, 1 μg/ml) for 24 h. Then 20 μL of MTT reagent (5 mg/mL) were added and incubated at 37 °C in the CO_2 incubator. After 4 h, 100 μL of DMSO was added. Absorbance was measured with spectrophotometer at 550 nm. The percent cell viability was calculated considering the untreated cells as 100% viability.

11. Cellular internalization of heparinoid-AuNPs.

Cell line	Growth Media
MDA-MB-468, MDA-MB-	Cells were grown at 37 °C in 5% CO_2 atmosphere in

231, T47D, MCF-7, SK-BR-3 and NIH-3T3	DMEM medium containing 10% fetal bovine serum and 0.1% streptomycin

All cells (2 x 10^4 cells per well) were seeded on 8-well chamber slides and allowed to grow for 24 h. Then heparinoid-AuNPs (10 µg/mL) was added in presence and absence of HB-EGF (20 ng/mL) for 4 h and 24 h respectively. Later the cells were washed three times with PBS buffer and placed in fresh media. Nuclei were stained with Hoechst 33342 reagent (2 µg/ mL). Fluorescence measurements were performed using excitation with an argon laser, *l* = 405, 488 & 561 nm, and the emission was collected at 450- 500 nm (for blue), 490-550 nm (for green) and 550- 650 nm (for red).

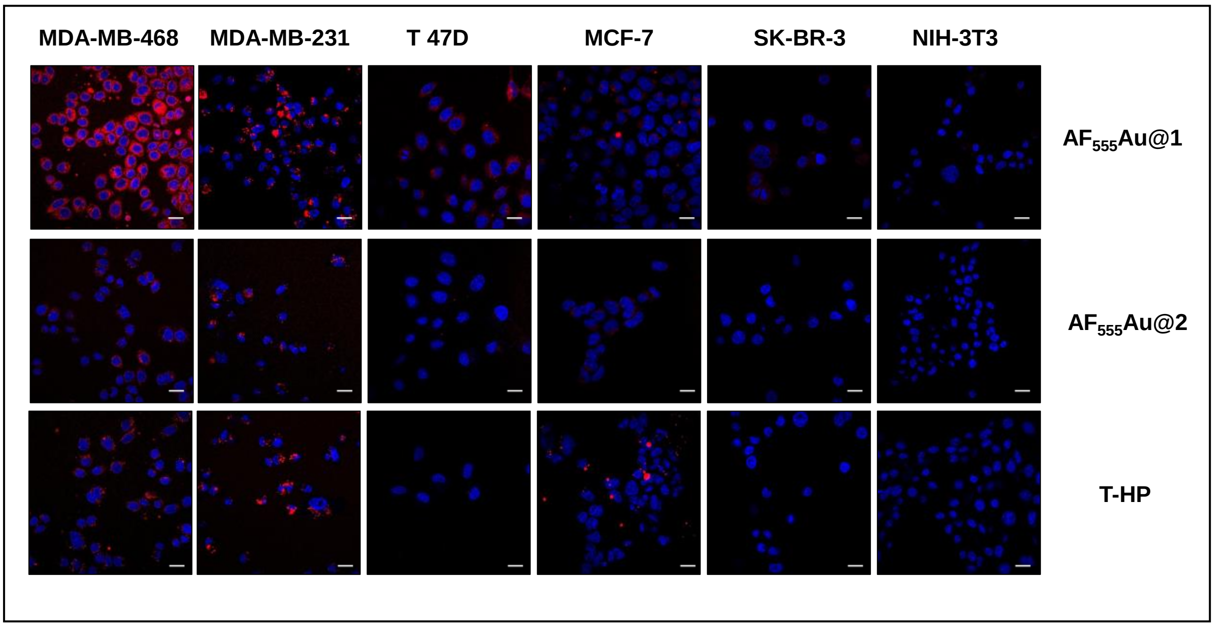

Cellular internalization at 24 h

Figure S5: Confocal images of nanoparticles internalization by different cells lines after 24 h (scale bar- 20 μm)

11.1 HB-EGF-Mediated uptake for MDA-MB-468

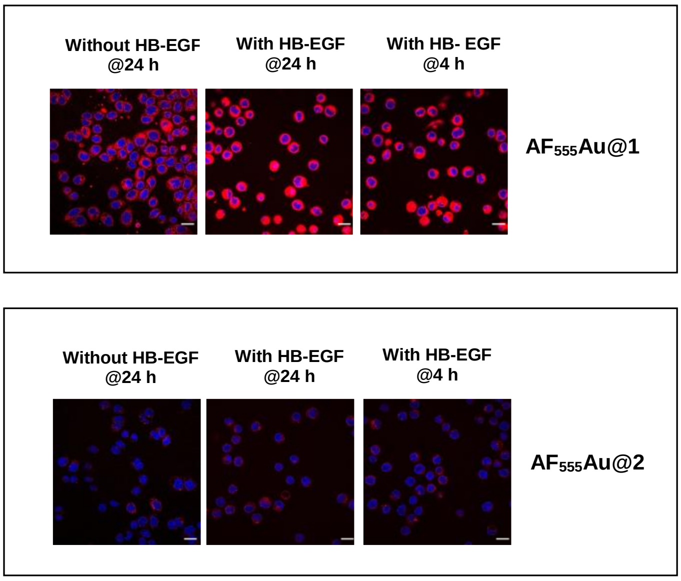

Figure S6: Cellular internalization of nanoparticles in presence of HB-EGF at 4 and 24 h. (scale bar: 20 *μ*m)

12. FACS analysis:

MDA-MB-468 cells (0.24 x 10^5) were seeded in 24 well plate and allowed to grow at 37 ^oC in 5 % CO_2 atmosphere till 70- 80 % confluency. After that heparinoid AuNPs (10 μg/ml) were added and incubated for 4 h. Upon incubation, wells were washed with PBS to remove the excess NPs. Finally, cells were trypsinized and resuspended in 0.5 ml PBS for FACS measurements. Cells without NPs were taken as a control and fluorescence with respect to that were measured to quantify the uptake in PE-A channel using yellow green (561 nm) laser.

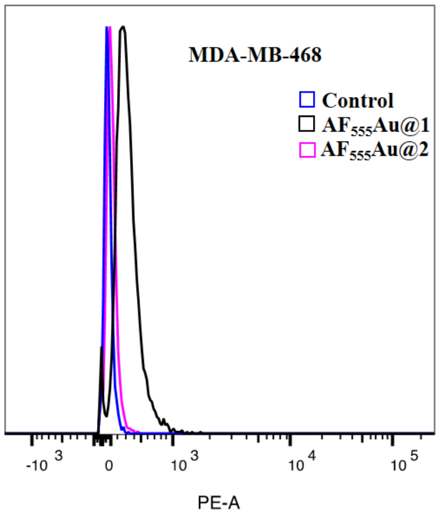

Figure S7: Histograms representing the cell uptake of **AF$_{555}$Au@1** and **AF$_{555}$Au@2** in MDA-MB-468 at 4 h.

13. Co-localization.

MDA-MB-468 (2 ×10^{4} cells/well) were seeded on 8-well chamber slides and incubated overnight. Cells were treated with **AF$_{555}$Au@1** (10 µg/mL) for 4 h. Cells were then washed twice with PBS buffer and treated with ER/ Mito/ Lyso tracker green at a concentration of 50 nM in PBS and incubated in dark at 37 °C for 30 min. The cells were treated with Hoechst 33342 to stain nuclei. The fluorescence of Hoechst 33342, green tracker and red tracker were excited with an argon laser at 405 nm 488 nm and 561 nm respectively, and the emission was collected at 450- 500 nm (for blue), 490- 550 nm (for green) and 550- 650 nm (for red)

13.1 ER Co-localization

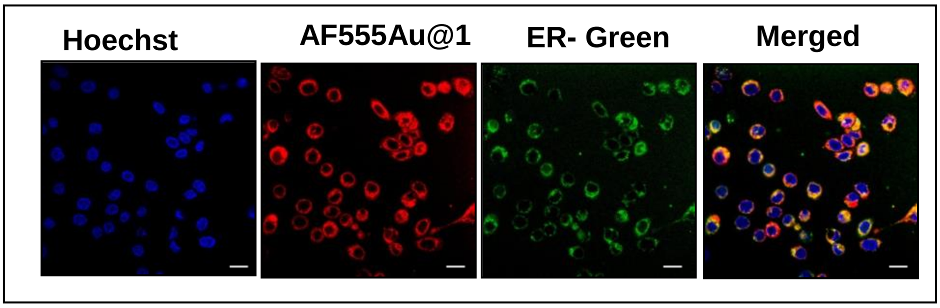

13.2 Lysosome Co-localization

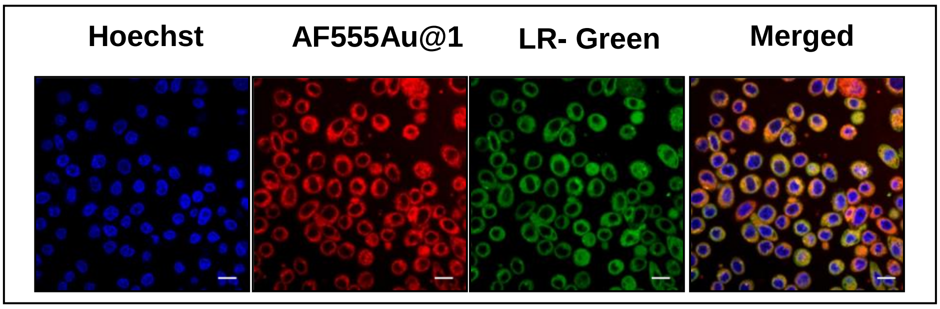

13.3 Mitochondria Co-localization

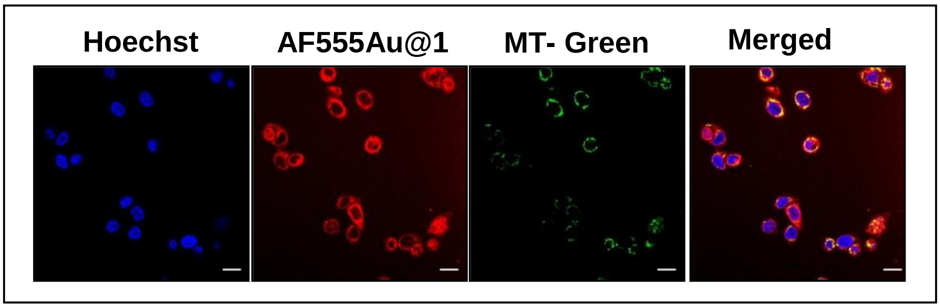

Figure S8: Cellular co-localization of **AF$_{555}$Au@1** in different cellular compartments. (scale bar: 20 μm)

14. Cellular internalization mechanism studies of AF$_{555}$Au@1 in MDA-MB-468

To prove the endocytosis pathway, we utilized EGFR inhibitor to prove the mechanism. We incubated MDA-MB-468 cells (2 $\times 10^4$ cells/well) in 8-well chamber slides and treated with NaN_3 (50 mM) for 30 min to deplete ATP, followed by the addition of **AF$_{555}$Au@1** for 4 h and imaged. To confirm EGFR mediated cellular internalization of nanoparticles, cells were treated with Gefitinib (EGFR inhibitor) at a concentration of 30 µM and incubated for 30 min h, followed by **AF$_{555}$Au@1** addition and imaged after 4 h.

15. 3D spheroid formation.

3D spheroids for MDA-MB-468 were formed using Matrigel (ECM Gel from Engelbreth-Holm-Swarm murine sarcoma) using manufacturer's protocol. Briefly, at first a monolayer of 100 % matrigel (~55 µL) was coated on 8-well chamber slides and kept for polymerization at 37 °C for 15 min. Next, mono-dispersed freshly trypsinized MDA-MB-468 (~4000 cells/well) in growth medium containing 2% matrigel was plated on top of the pure matrigel layer and incubated at 37 °C for 7-8 days. Growth Medium was replaced every 2nd day. After spheroid formation heparinoid-AuNPs (50 µg/mL) were added and incubated for 4 h. Later, the cells were washed three times with PBS buffer and placed in fresh media. Nuclei were stained with Hoechst 33342 reagent (2 µg/mL). For co-culture experiments cells in the ratio of (2:1) of MDA-MB-468 and GFP stable NIH-3T3 were added and allowed to grow till 5 days for spheroids formation. Fluorescence measurements were performed using excitation with an argon laser, *l* = 405nm, 488 nm and 516 nm with the emission collected through 403–452 nm , 490-540 nm and 550–650 nm filters.

16. Abbreviations:

PTSA	p-Toluenesulfonic Acid
DMAP	4- Dimethylaminopyridine
TBDPS-Cl	tert-Butyldiphenylchlorosilane
NIS	*N*- Iodosuccinimide
TMSOTf	Trimethylsilyl trifluoromethanesulfonate

TfOH	Trifluoromethanesulfonic acid
TEMPO	2,2,6,6-tetramethylpiperidin-1-yloxyl
BAIB	[bis(acetoxyiodo)]benzene
NHS	*N*-Hydroxysuccinimide
DDQ	2, 3-Dichloro-5, 6-dicyano-1, 4-benzoquinone
RT	Room Temperature

17. References:

1. (a) P. Jain, C. D. Shanthamurthy, S. L. Ben-Arye, R. J. Woods, R. Kikkeri, V. Padler-Karavani, ***Chem Sci.*** *(Just Accepted)* ***https://doi.org/10.1039/D0SC05862A***; (b) S. Anand, S. Mardhekar, R. Raigawali, N. Mohanta, P. Jain, C. D. Shanthamurthy, B. Gnanaprakasam and R. Kikkeri, *Org. Lett.*, 2020, **22**, 3402-3406.

18. ^{1}H and ^{13}C NMR:

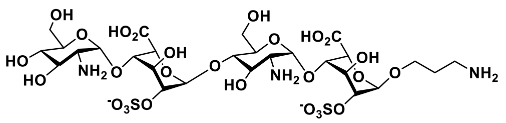

1

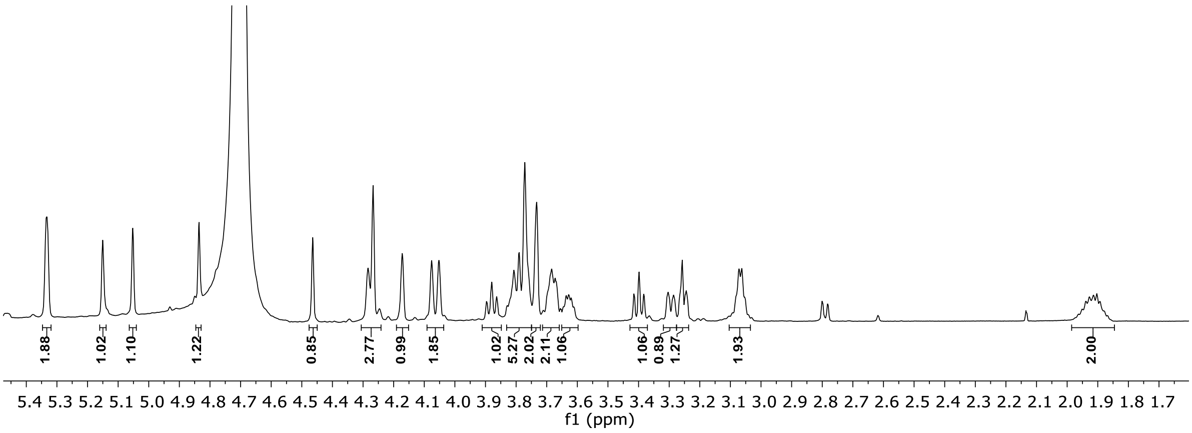

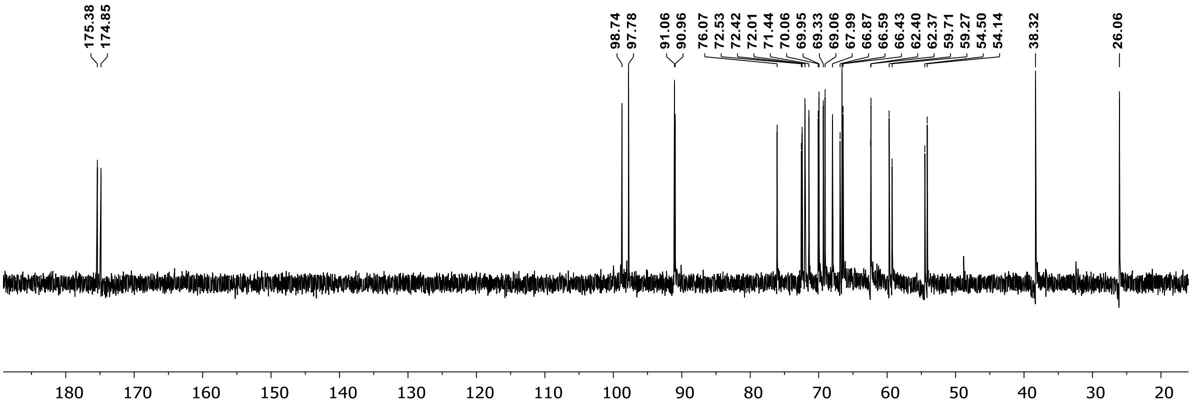

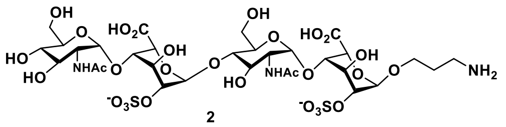
OH
HO
HO
NHAc
HO2C
OH
-O3SO
OH
HO
NHAc
HO2C
OH
-O3SO
NH2

2

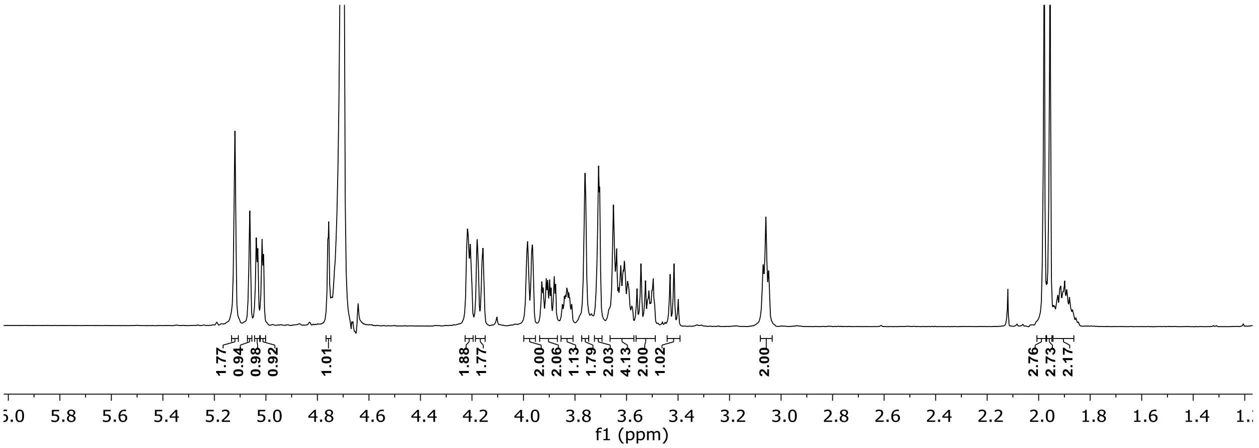
1.77
0.94
0.98
0.92
1.01
1.88
1.77
2.00
2.06
1.13
1.79
2.03
4.13
2.00
1.02
2.00
2.76
2.73
2.17
6.0 5.8 5.6 5.4 5.2 5.0 4.8 4.6 4.4 4.2 4.0 3.8 3.6 3.4 3.2 3.0 2.8 2.6 2.4 2.2 2.0 1.8 1.6 1.4 1.2
f1 (ppm)

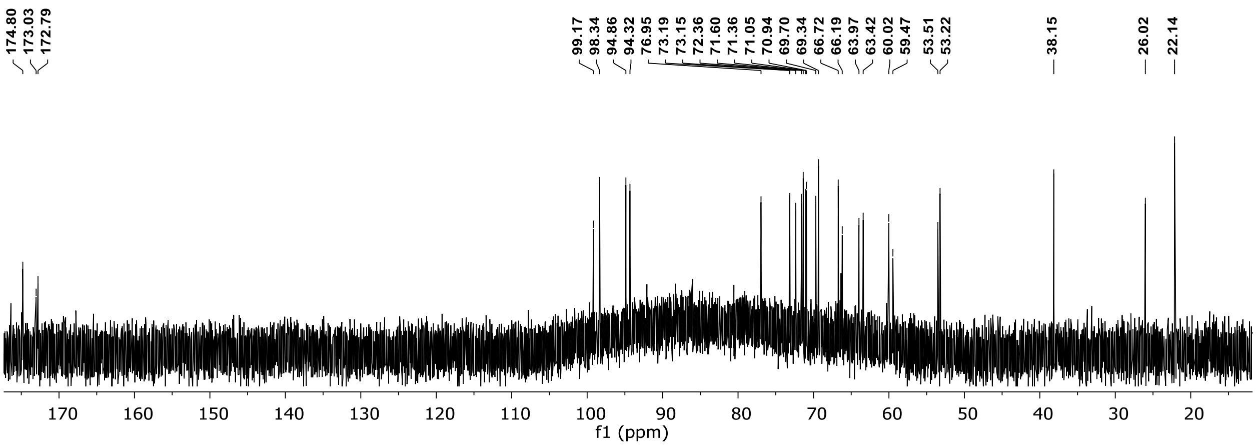
174.80
173.03
172.79
99.17
98.34
94.86
94.32
76.95
73.19
73.15
72.36
71.60
71.36
71.05
70.94
69.70
69.34
66.72
66.19
63.97
63.42
60.02
59.47
53.51
53.22
38.15
26.02
22.14
170 160 150 140 130 120 110 100 90 80 70 60 50 40 30 20
f1 (ppm)

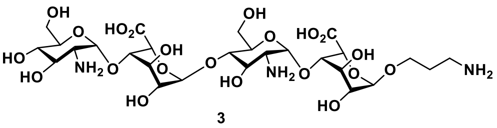
OH
HO
HO
NH2
HO2C
OH
O
HO
NH2
NH2

3

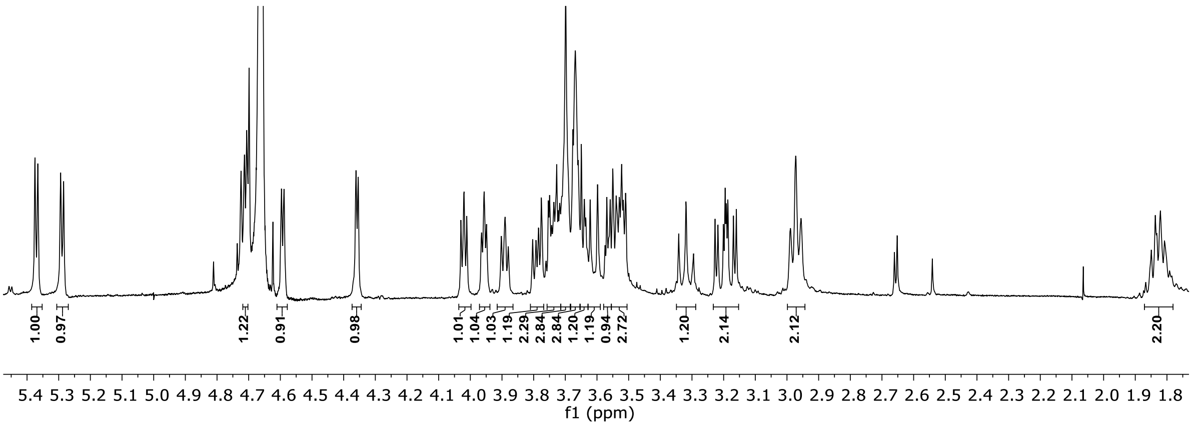
1.00
0.97
1.22
0.91
0.98
1.01
1.04
1.03
1.19
2.29
2.84
2.84
1.20
1.19
0.94
2.72
1.20
2.14
2.12
2.20
5.4 5.3 5.2 5.1 5.0 4.9 4.8 4.7 4.6 4.5 4.4 4.3 4.2 4.1 4.0 3.9 3.8 3.7 3.6 3.5 3.4 3.3 3.2 3.1 3.0 2.9 2.8 2.7 2.6 2.5 2.4 2.3 2.2 2.1 2.0 1.9 1.8
f1 (ppm)

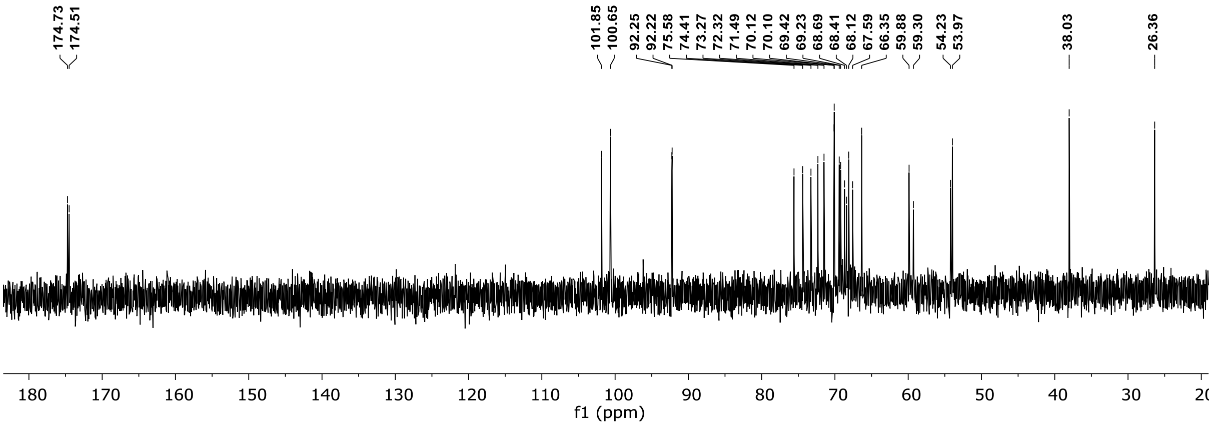
174.73
174.51
101.85
100.65
92.25
92.22
75.58
74.41
73.27
72.32
71.49
70.12
70.10
69.42
69.23
68.69
68.41
68.12
67.59
66.35
59.88
59.30
54.23
53.97
38.03
26.36
180 170 160 150 140 130 120 110 100 90 80 70 60 50 40 30
f1 (ppm)

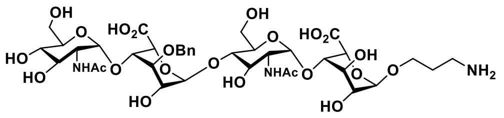
OH
HO₂C
OBn
HO
NHAc
HO
HO
O
OH
HO₂C
OH
NHAc
HO
HO
NH₂

4

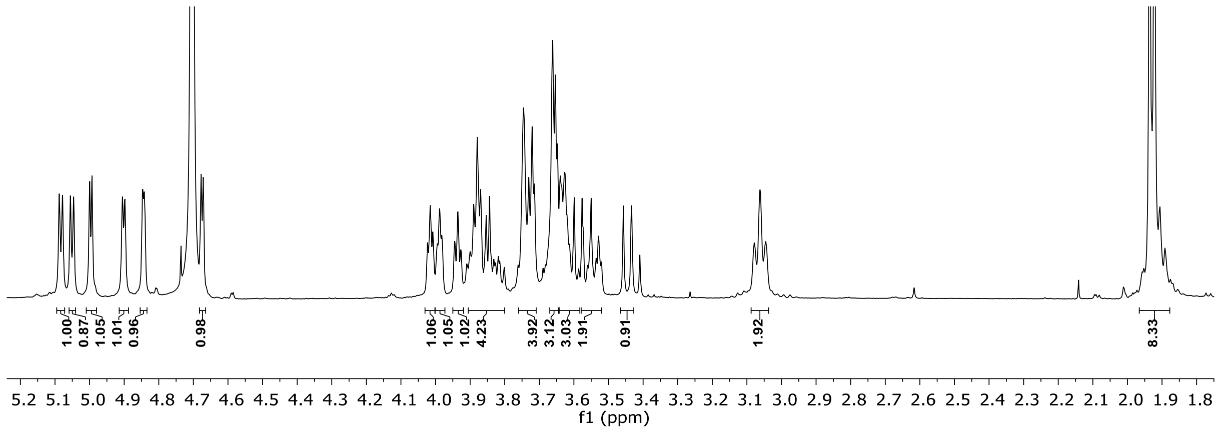
1.00
0.87
1.05
1.01
0.96
0.98
1.06
1.05
1.02
4.23
3.92
3.12
3.03
1.91
0.91
1.92
8.33
5.2 5.1 5.0 4.9 4.8 4.7 4.6 4.5 4.4 4.3 4.2 4.1 4.0 3.9 3.8 3.7 3.6 3.5 3.4 3.3 3.2 3.1 3.0 2.9 2.8 2.7 2.6 2.5 2.4 2.3 2.2 2.1 2.0 1.9 1.8
f1 (ppm)

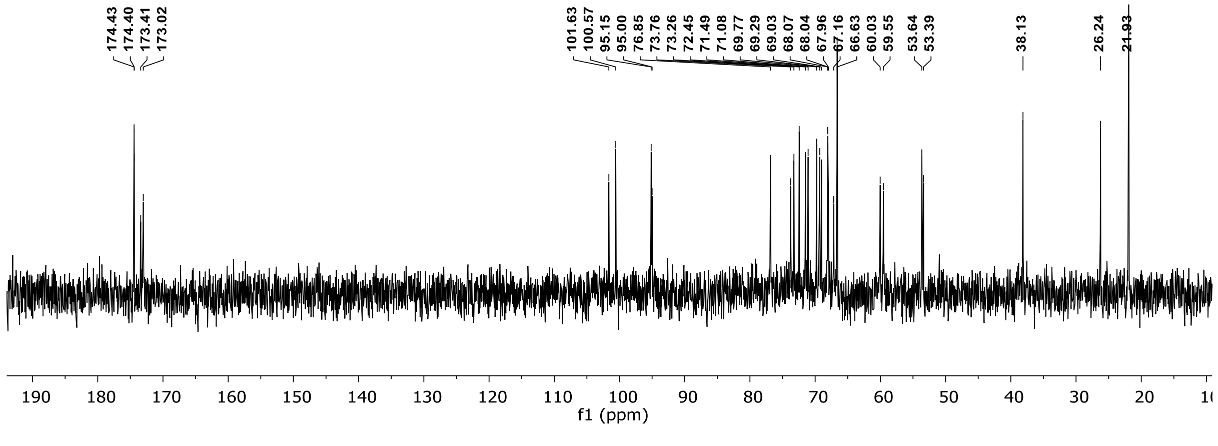
174.43
174.40
173.41
173.02
101.63
100.57
95.15
95.00
76.85
73.76
73.26
72.45
71.49
71.08
69.77
69.29
69.03
68.07
68.04
67.96
67.16
66.63
60.03
59.55
53.64
53.39
38.13
26.24
21.93
190 180 170 160 150 140 130 120 110 100 90 80 70 60 50 40 30 20
f1 (ppm)

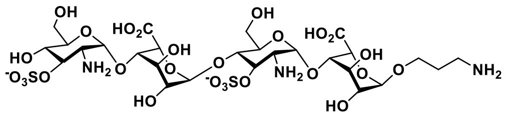
OH
HO₂C
OH
HO
O
NH₂
-O₃SO
HO
HO₂C
OH
NH₂
HO
NH₂

5

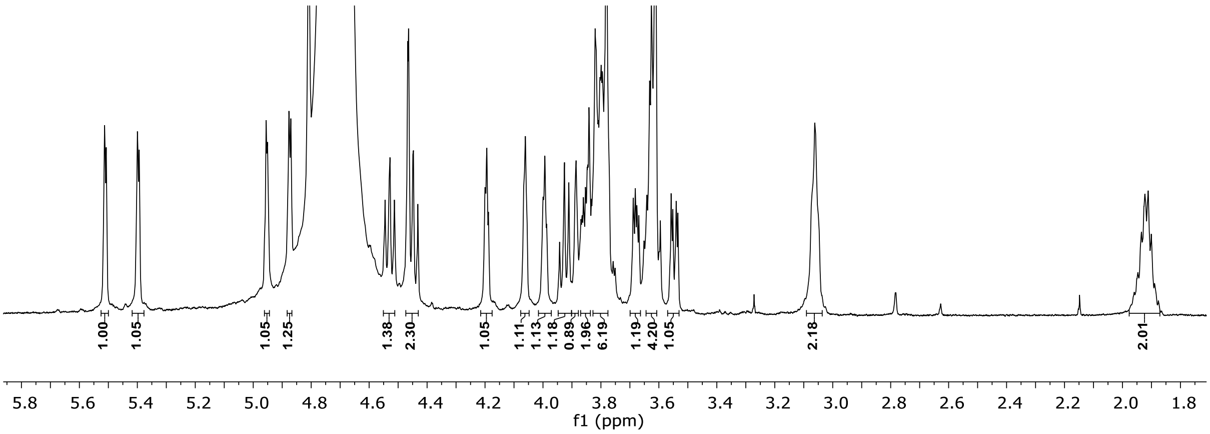
1.00
1.05
1.05
1.25
1.38
2.30
1.05
1.11
1.13
1.18
0.89
1.96
6.19
1.19
4.20
1.05
2.18
2.01
5.8 5.6 5.4 5.2 5.0 4.8 4.6 4.4 4.2 4.0 3.8 3.6 3.4 3.2 3.0 2.8 2.6 2.4 2.2 2.0 1.8
f1 (ppm)

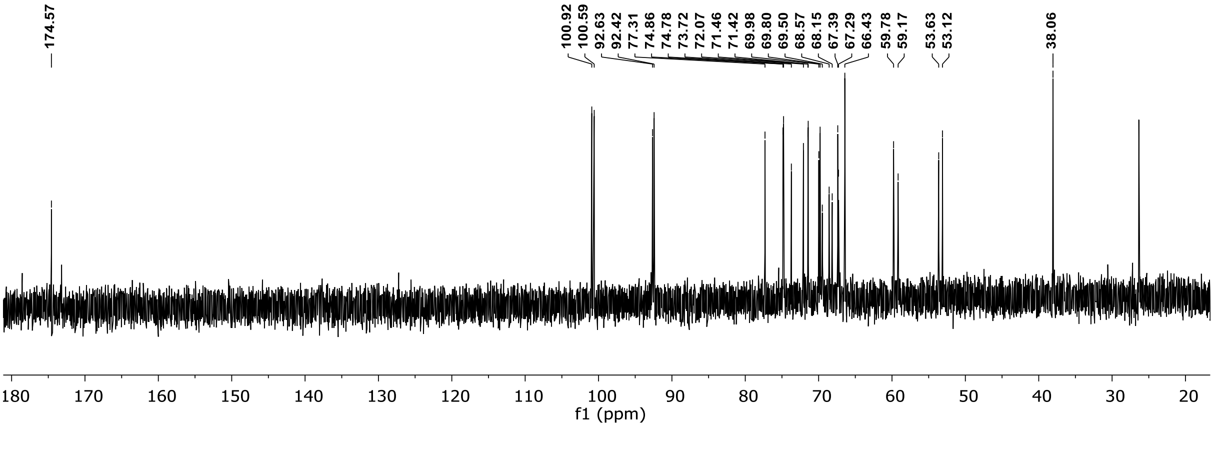
174.57
100.92
100.59
92.63
92.42
77.31
74.86
74.78
73.72
72.07
71.46
71.42
69.98
69.80
69.50
68.57
68.15
67.39
67.29
66.43
59.78
59.17
53.63
53.12
38.06
180 170 160 150 140 130 120 110 100 90 80 70 60 50 40 30 20
f1 (ppm)

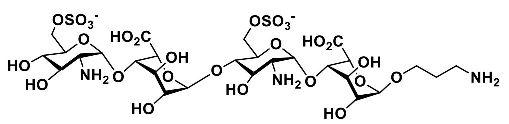
OSO3-
HO2C
OH
HO
NH2
O
HO
OSO3-
HO2C
OH
HO
NH2
O
HO
NH2

6

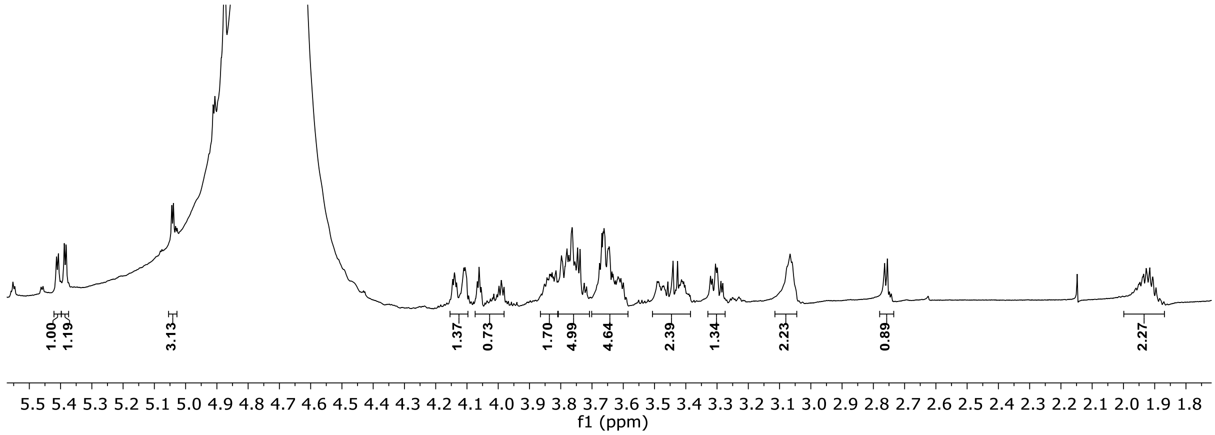
1.00
1.19
3.13
1.37
0.73
1.70
4.99
4.64
2.39
1.34
2.23
0.89
2.27
5.5 5.4 5.3 5.2 5.1 5.0 4.9 4.8 4.7 4.6 4.5 4.4 4.3 4.2 4.1 4.0 3.9 3.8 3.7 3.6 3.5 3.4 3.3 3.2 3.1 3.0 2.9 2.8 2.7 2.6 2.5 2.4 2.3 2.2 2.1 2.0 1.9 1.8
f1 (ppm)

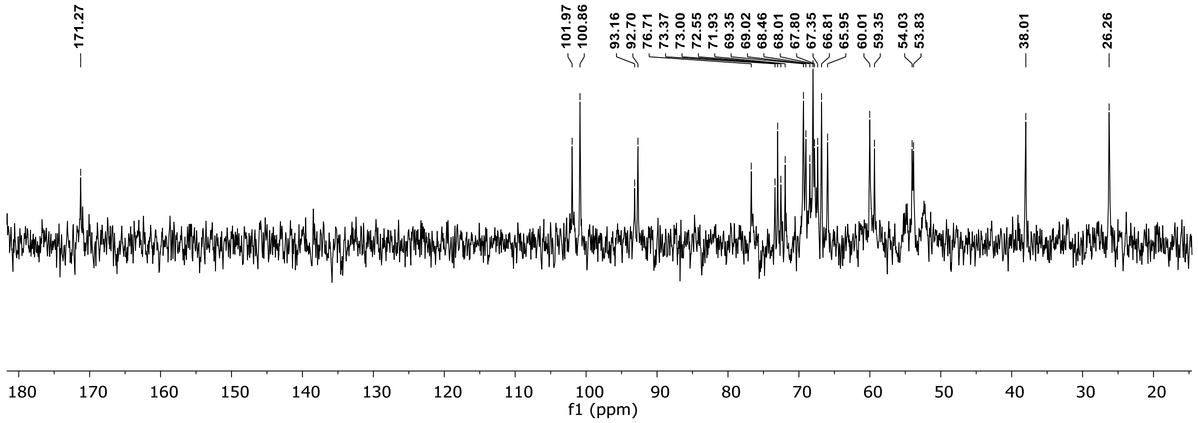
171.27
101.97
100.86
93.16
92.70
76.71
73.37
73.00
72.55
71.93
69.35
69.02
68.46
68.01
67.80
67.35
66.81
65.95
60.01
59.35
54.03
53.83
38.01
26.26
180 170 160 150 140 130 120 110 100 90 80 70 60 50 40 30 20
f1 (ppm)

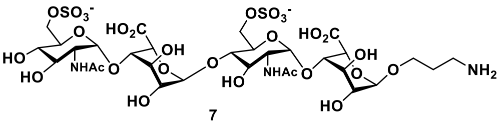
OSO3-
HO2C
OH
O
HO
HO
NHAc
O
HO
O
OSO3-
O
HO2C
OH
HO
NHAc
O
O
O
HO
NH2

7

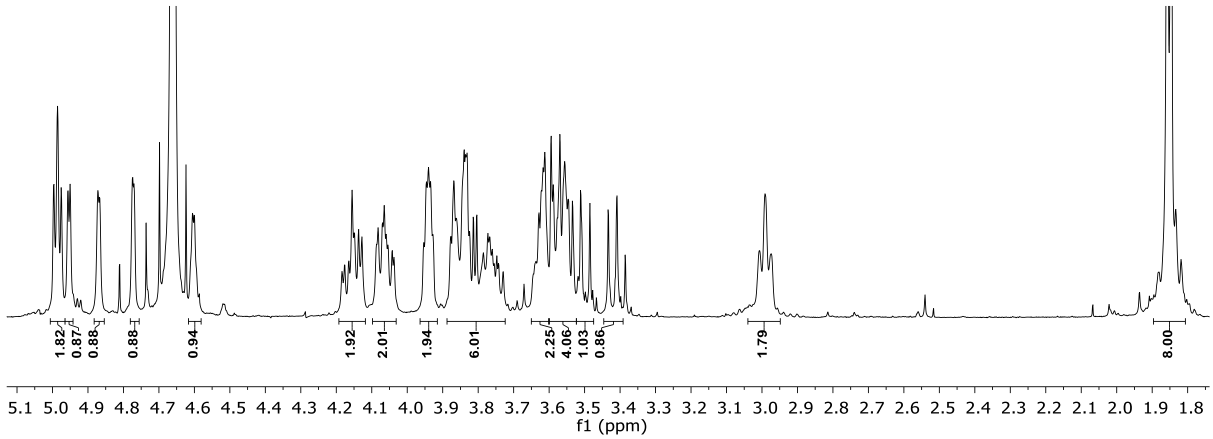
1.82
0.87
0.88
0.88
0.94
1.92
2.01
1.94
6.01
2.25
4.06
1.03
0.86
1.79
8.00
5.1 5.0 4.9 4.8 4.7 4.6 4.5 4.4 4.3 4.2 4.1 4.0 3.9 3.8 3.7 3.6 3.5 3.4 3.3 3.2 3.1 3.0 2.9 2.8 2.7 2.6 2.5 2.4 2.3 2.2 2.1 2.0 1.9 1.8
f1 (ppm)

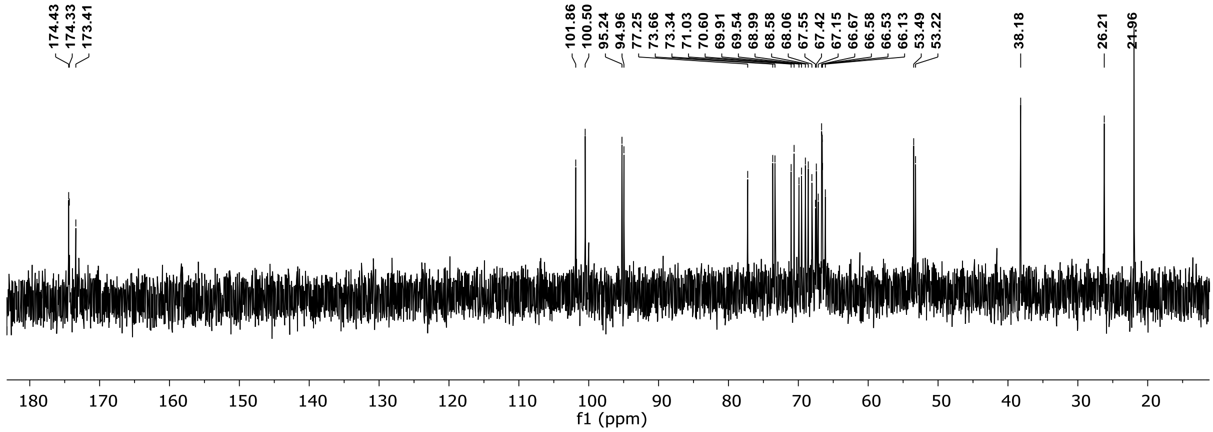
174.43
174.33
173.41
101.86
100.50
95.24
94.96
77.25
73.66
73.34
71.03
70.60
69.91
69.54
68.99
68.58
68.06
67.55
67.42
67.15
66.67
66.58
66.53
66.13
53.49
53.22
38.18
26.21
21.96
180 170 160 150 140 130 120 110 100 90 80 70 60 50 40 30 20
f1 (ppm)

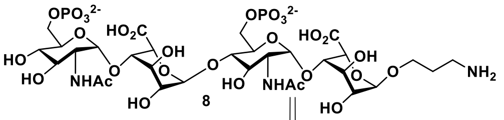
OPO3 2-
HO2C
OH
HO
HO
NHAc
O
HO
OPO3 2-
HO2C
OH
HO
NHAc
O
HO
NH2

8

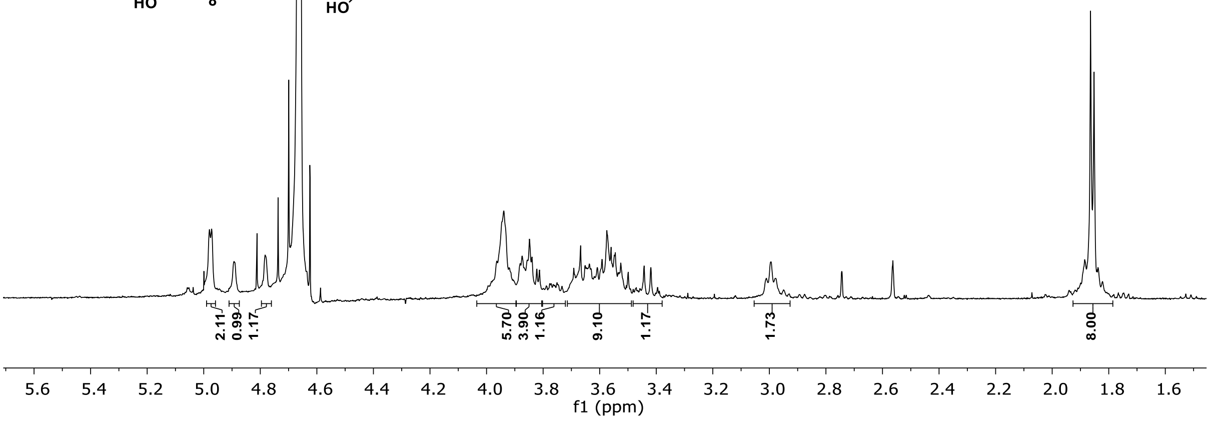
2.11
0.99
1.17
5.70
3.90
1.16
9.10
1.17
1.73
8.00
5.6 5.4 5.2 5.0 4.8 4.6 4.4 4.2 4.0 3.8 3.6 3.4 3.2 3.0 2.8 2.6 2.4 2.2 2.0 1.8 1.6
f1 (ppm)

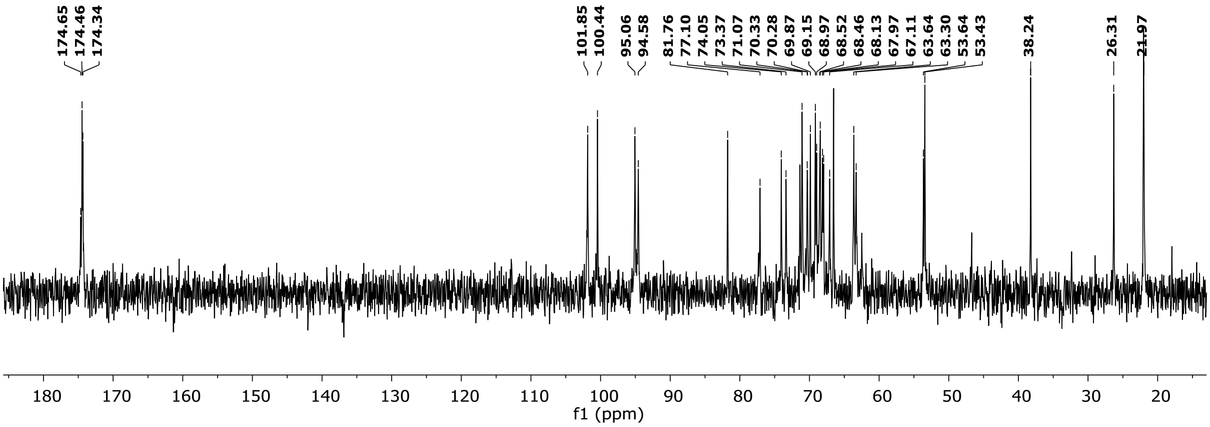
174.65
174.46
174.34
101.85
100.44
95.06
94.58
81.76
77.10
74.05
73.37
71.07
70.33
70.28
69.87
69.15
68.97
68.52
68.46
68.13
67.97
67.11
63.64
63.30
53.64
53.43
38.24
26.31
21.97
180 170 160 150 140 130 120 110 100 90 80 70 60 50 40 30 20
f1 (ppm)

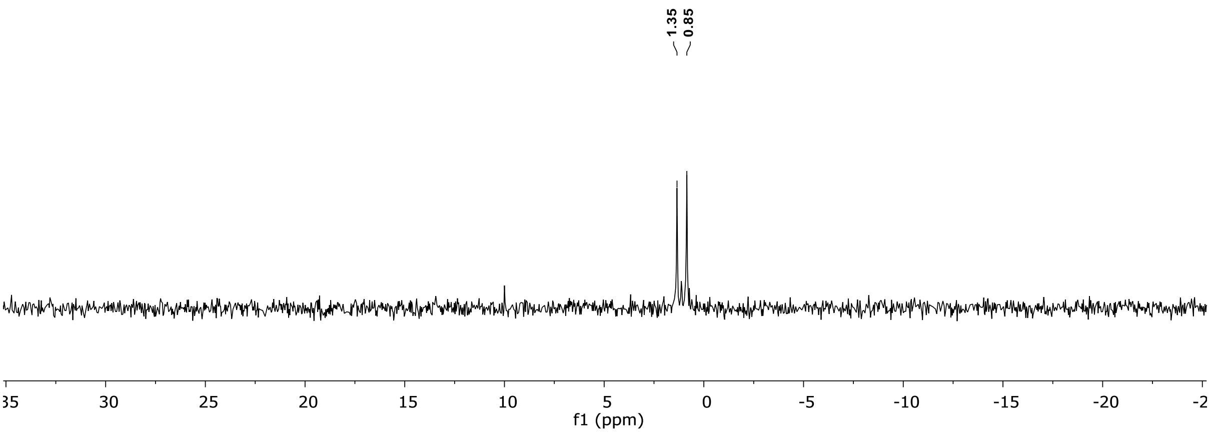
1.35
0.85
35 30 25 20 15 10 5 0 -5 -10 -15 -20 -2
f1 (ppm)
